# Low- and High-Pathogenic Avian Influenza H5 and H7 Spread Risk Assessment Within and Between Australian Commercial Chicken Farms

**DOI:** 10.3389/fvets.2018.00063

**Published:** 2018-04-09

**Authors:** Angela Bullanday Scott, Jenny-Ann L. M. L. Toribio, Mini Singh, Peter Groves, Belinda Barnes, Kathryn Glass, Barbara Moloney, Amanda Black, Marta Hernandez-Jover

**Affiliations:** ^1^Sydney School of Veterinary Science, Faculty of Science, University of Sydney, Sydney, NSW, Australia; ^2^Quantitative Sciences, Department of Agriculture and Water Resources, Canberra, ACT, Australia; ^3^College of Medicine, Biology and Environment, Australian National University, Canberra, ACT, Australia; ^4^New South Wales Department of Primary Industries, Orange, NSW, Australia; ^5^Graham Centre for Agricultural Innovation, School of Animal and Veterinary Sciences, Charles Sturt University and New South Wales Department of Primary Industries, Wagga Wagga, NSW, Australia; ^6^School of Animal and Veterinary Sciences, Charles Sturt University, Wagga Wagga, NSW, Australia

**Keywords:** avian influenza, Australia, commercial chickens, H5, H7, scenario trees, partial consequence assessment, spread

## Abstract

This study quantified and compared the probability of avian influenza (AI) spread within and between Australian commercial chicken farms via specified spread pathways using scenario tree mathematical modeling. Input values for the models were sourced from scientific literature, expert opinion, and a farm survey conducted during 2015 and 2016 on Australian commercial chicken farms located in New South Wales (NSW) and Queensland. Outputs from the models indicate that the probability of no establishment of infection in a shed is the most likely end-point after exposure and infection of low-pathogenic avian influenza (LPAI) in one chicken for all farm types (non-free range meat chicken, free range meat chicken, cage layer, barn layer, and free range layer farms). If LPAI infection is established in a shed, LPAI is more likely to spread to other sheds and beyond the index farm due to a relatively low probability of detection and reporting during LPAI infection compared to high-pathogenic avian influenza (HPAI) infection. Among farm types, the median probability for HPAI spread between sheds and between farms is higher for layer farms (0.0019, 0.0016, and 0.0031 for cage, barn, and free range layer, respectively) than meat chicken farms (0.00025 and 0.00043 for barn and free range meat chicken, respectively) due to a higher probability of mutation in layer birds, which relates to their longer production cycle. The pathway of LPAI spread between sheds with the highest average median probability was spread via equipment (0.015; 5–95%, 0.0058–0.036) and for HPAI spread between farms, the pathway with the highest average median probability was spread via egg trays (3.70 × 10^−5^; 5–95%, 1.47 × 10^−6^–0.00034). As the spread model did not explicitly consider volume and frequency of the spread pathways, these results provide a comparison of spread probabilities per pathway. These findings highlight the importance of performing biosecurity practices to limit spread of the AI virus. The models can be updated as new information on the mechanisms of the AI virus and on the volume and frequency of movements shed-to-shed and of movements between commercial chicken farms becomes available.

## Introduction

The risk of low-pathogenic avian influenza (LPAI) virus spread in Australia is initially dependent on the risk of exposure of commercial chicken farms in this country to LPAI, which has been quantified for New South Wales by Scott et al. (1). After exposure to the virus, the risk of spread is then dependent on infection of the chicken with the virus and establishment of the virus within the flock (2–4). Once established in one flock, LPAI spread within farms (between sheds) and between farms can occur. LPAI infection can be associated with no clinical signs but a range of clinical illness in birds including respiratory disease can also be seen, thereby leading to production losses and decreased welfare (2, 5). For infections with H5 and H7 LPAI viruses, with further virus spread and the subsequent increasing number of infected birds, there is a greater possibility of mutation of the virus to high-pathogenic avian influenza (HPAI). HPAI has very high morbidity and mortality rates in gallinaceous poultry (up to 100%) (5). If mutation does occur, the risk of HPAI spread within and between farms must then be considered.

Factors influencing the success of LPAI or HPAI spread depend heavily on biosecurity actions put into place on the farm. Previous modeling work suggest that bird pickup trucks and feed trucks that move between farms and human movements between sheds were pathways associated with the highest risk of spread of AI. Emphasis to ensure good biosecurity practices associated with these pathways, such as vehicle disinfection and footbaths, was therefore made (6, 7). The timeliness of detection of clinical signs of infected flocks by farmers also plays a significant role in limiting spread of the disease. If the appropriate authority figures are contacted by farmers promptly, management practices can be put into place to limit spread of the virus both within and between farms (2, 8). This is supported by several previous mathematical modeling studies that revealed a reduction in the probability of AI spread to other farms if detection and reporting occurs earlier rather than later in the outbreak and if the detection threshold is lowered or frequent sampling occurs on high-risk farms (9–11).

All seven HPAI outbreaks in Australia to date have had only commercial chicken farms as the index farms; including commercial layer or meat chicken farms, with two outbreaks involving meat chicken breeder farms. Four of the seven HPAI outbreaks involved spread from the index farm to affect the nearby farms (12, 13). In addition, surveillance found evidence of LPAI infection among duck farms in the vicinity for two of the seven HPAI outbreaks, suggesting initial LPAI spread with subsequent mutation (14, 15). The focus on commercial chicken farms in this study is due to the comparatively small threat posed by non-commercial chicken farms to the Australian poultry industry. There is limited contact between non-commercial and commercial chicken farms in Australia. In addition, AI detection on non-commercial chicken farms, as did occur with three of the 12 LPAI cases detected in this country to date, has little impact on the industry, market, and consumers due to the small number of birds to destroy (14–16).

The pathways of spread in the past Australian HPAI outbreaks were suspected based on epidemiological investigations; examples identified include common dead bird pick up and egg transport vehicles among the affected farms (13, 17, 18). However, it is currently unknown for the Australian context which pathways are most likely to cause spread, whether particular farm types are at more risk of spread than others, and the influence biosecurity practices will have on spread. Thus there is a need to quantify and compare the probability of both LPAI and HPAI spread for all types of Australian commercial chicken enterprises, i.e., cage, barn, and free range systems of both layer and meat chicken farms. Further, there is a need to quantify the effect of on-farm preventive actions that can mitigate the risk and impact of future AI outbreak occurrences in Australia.

In response to these needs, the aim of this study was first to estimate the probability of infection and establishment of LPAI virus after one chicken is exposed to the virus using results obtained from Scott et al. (1). Then, potential pathways for LPAI and HPAI spread between sheds and farms on all types of Australian commercial chicken enterprises were identified. A partial consequence assessment was then performed to estimate and compare the probabilities of LPAI and of HPAI spread between sheds and farms with particular focus on the differences in spread via the investigated pathways, without explicit consideration of pathway volume and frequency as insufficient information was available to incorporate consideration of these in this study. Comparison of study results will inform understanding of the most influential pathways of spread of LPAI and HPAI, and of any differences between farm types if these exist. This new knowledge can direct thinking about on-farm biosecurity practices that can be put into place to reduce the potential for AI spread.

## Materials and Methods

### Risk Assessment Model

The overall study used the World Organisation of Animal Health (OIE) risk analysis framework (19) to conduct an exposure and partial consequence assessment in relation to AI for Australian commercial chicken farms. The exposure assessment considered the potential pathways by which chickens situated in a commercial layer or meat chicken farm can be exposed to avian influenza (AI) virus from wild birds. This assessment can be found in the study by Scott et al. (1). The current study focused on a partial consequence assessment, where the risk of spread was determined but the level of consequences following spread was not measured. This assessment considered the pathways by which these viruses can spread between sheds on the same farm and from one farm to other farms. The probability of these pathways occurring was calculated. Such pathways were portrayed using scenario trees (20) and developed using Microsoft Excel (PC/Windows 7, 2010). The probabilities were estimated using Monte Carlo stochastic simulation modeling using the program @RISK 7.0 (Palisade Corporation, USA). Each simulation consisted of 50,000 iterations sampled using the Latin hypercube method with a fixed random seed of one.

### Data Sources

Most of the input values used in this model were parameterized using data collected from a survey on commercial chicken farms in Australia (8, 21). This study defined commercial layer farms as those with more than 1,000 birds, and commercial meat chicken farms as those with more than 25,000 birds. It involved a comprehensive on-farm interview with farmers including questions related to farm management, biosecurity practices, and wild bird presence. In addition, input values were also obtained from scientific literature. An expert opinion workshop was also held to obtain input values that were largely unknown or undescribed in the scientific literature (22).

### Survey on Commercial Layer and Meat Chicken Farms in the Sydney Basin Region and South East Queensland

A survey was conducted from mid-2015 with on-farm interviews on 73 commercial chicken farms; nine cage layer, 9 barn layer, 25 free range layer, 15 non-free range meat chicken, and 15 free range meat chicken farms (8, 21). The farms were located in the Sydney basin region in New South Wales (NSW) and in South East Queensland. The Sydney basin region was selected due to the high concentration of both layer and meat chicken farms in this area. However, in this region, free range meat chicken farms are all owned by one of the two large privately owned meat chicken companies in Australia. Therefore, additional farm visits to South East Queensland were conducted to gain more representative data of privately owned meat chicken companies in Australia. The interviews with the farm manager or farm owner involved a comprehensive questionnaire with questions relating to biosecurity practices performed on farm, wild bird and animal presence, general farm information, and farm management. A greater proportion of layer farms and of free range farms were surveyed due to the greater perceived risk of AI occurrence on these farm types. Further details on the survey methodology, including the region and farm selection, questionnaire development, and conduct of the on-farm interviews can be found in the study by Scott et al. (21).

### Expert Opinion

Due to many unknowns related to the AI virus, an expert elicitation process was conducted in late 2015 to help inform the parameters of mutation from LPAI to HPAI and farm-to-farm spread pathways; the shed-to-shed spread pathways were informed from a combination of scientific literature and the farm survey. The elicitation process used a modified Delphi technique to gather the information, based on a four-step elicitation process. The process involved the experts completing a questionnaire individually, followed by a discussion of the results at a workshop, and then a reassessment of the questionnaire answers after the workshop. A total of 10 experts who had varying levels of expertise related to the poultry industry, wild bird behavior, and AI virus characteristics, participated in the process. The experts were selected based on their experience in the Australian poultry industries including involvement in the management of HPAI outbreaks in Australia or overseas as well as knowledge on the AI virus and wild birds. The questionnaire included 39 probability questions, and experts were asked to provide a most likely, minimum and maximum estimates of the probabilities and their level of confidence on their estimates. Pert distributions were used to obtain individual expert estimates for each question. The second round of estimates for each question for all experts was then combined using a weighting factor depending on their respective level of expertise relevant to each question, in a discrete distribution. More details on the expert elicitation process and the outcomes of the study can be found in the study by Singh et al. (22).

### Statistical Analysis

The statistical program JMP^®^ was used (© 2012 SAS Institute Inc., Cary, NC, USA) to conduct one-way analysis of variance (ANOVA) to analyze the differences between the outcome probabilities from the models for different farm types. The outcome probabilities compared using ANOVA were the outcome probability from 1,000 iterations of each pathway endpoint of the spread scenario tree model simulation for each farm type with each iteration reflecting the situation for one farm at any point in time. A *p*-value of <0.05 was used to determine statistical significance in these analyses.

### Partial Consequence (Spread) Assessment

The partial consequence assessment investigates the pathways of AI virus spread after one bird has been exposed to the virus at any point in time. It provides a comparison of spread probabilities between pathways; however, the volume and frequency of each pathway occurring were not explicitly considered. For shed-to-shed spread, there is consideration of the proportion of farms that perform or have these pathways present in combination with the survival of the virus on these pathways. For farm-to-farm spread, it was assumed that variation between pathways in volume and frequency and in virus survival was considered by experts. From the assumed LPAI exposure of one bird, spread first depends on infection of this bird, and this probability differs between direct or indirect exposure. In addition, spread depends on establishment of the virus within the shed after infection of one individual, which is influenced by the subtype of the virus. Both LPAI and HPAI spread are assessed, where the probability of H5/H7 mutation from LPAI to HPAI is also considered after establishment within a flock. The end-points of this model are exclusive of one another and are as follows: (1) no establishment of the infection; (2) limited LPAI spread; (3) limited HPAI spread; (4) LPAI spread; and (5) HPAI spread.

Limited spread is defined as the spread that would occur even when infection is detected and reported by the farmer. In this situation, although it is assumed that control measures will be put into place to restrict further spread of the virus, spread prior to detection and reporting would be likely to occur due to the routine large volume of activities between both sheds and farms. Supporting this assumption, the number of days required for detection and reporting was estimated using an index function on Microsoft Excel, resulting in a time period of at least 70 days from infection of the first chicken with LPAI to establishment, detection and reporting by the farmer for all farm types. This estimation considered a reproduction number (*R*) of 1.35, the proportion of birds showing clinical signs, the shed size, and the percentage threshold for LPAI detection and reporting. The calculation of R and the proportion of birds showing clinical signs are presented in the description of the *Establishment of LPAI after infection in one chicken* node in the Supplementary Material. The shed size and percentage threshold for LPAI detection and reporting differ for each farm type and are described by Scott et al. (21). If there is no detection and reporting, the potential pathways by which LPAI and HPAI can spread between sheds and between farms are evaluated for each farm type.

**Figure 1:**
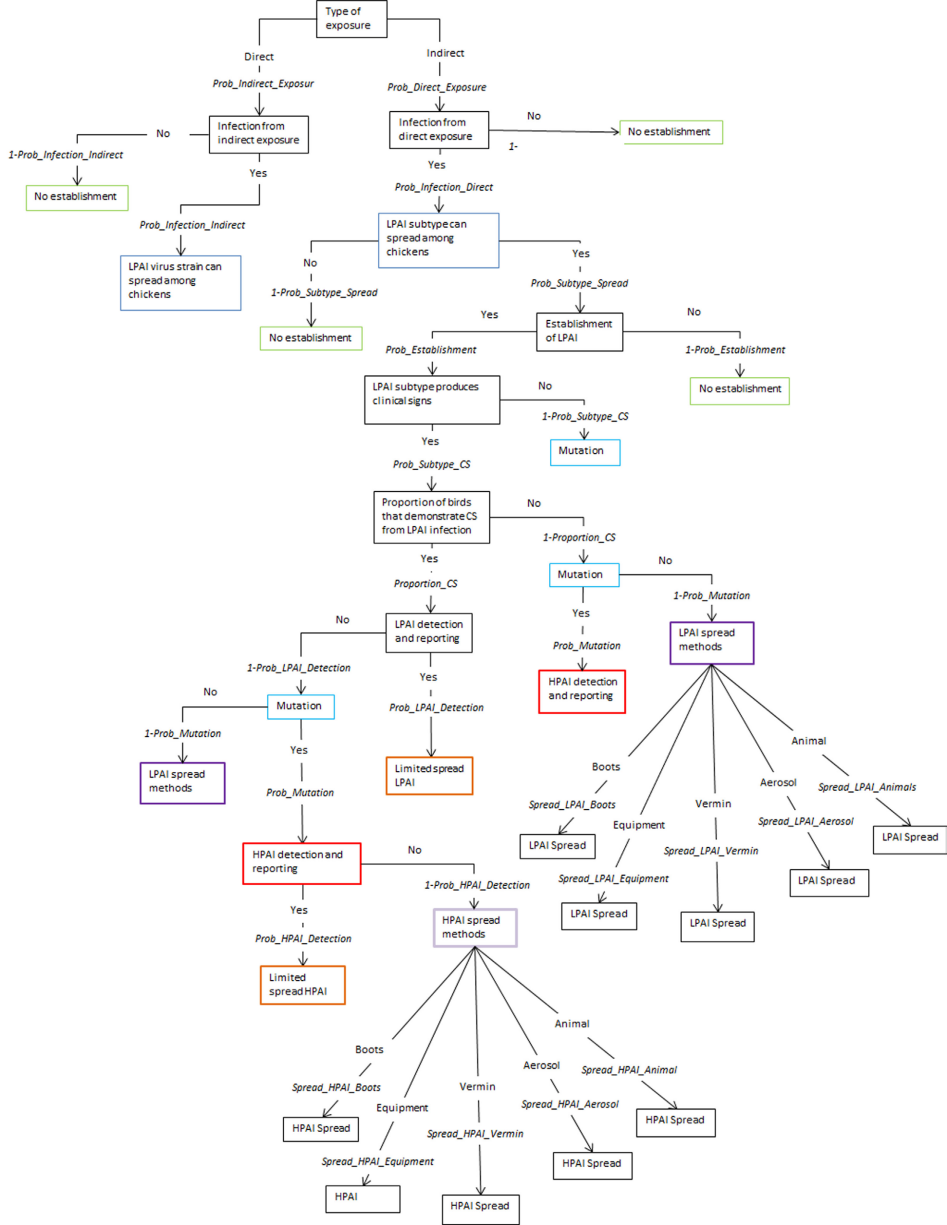
Scenario tree representing the spread pathways of low-pathogenic and high-pathogenic avian influenza (LPAI and HPAI) viruses between sheds for Australian commercial layer and meat chicken farms. (*Prob_Indirect_Exposure, probability of indirect exposure of LPAI virus to a commercial chicken; Prob_Direct_Exposure, probability of direct exposure of LPAI virus to a commercial chicken; Prob_Infection_Indirect, probability of infection of LPAI after indirect exposure; Prob_Infection_Direct, probability of infection of LPAI after direct exposure; Prob_Subtype_Spread, probability that the H5/H7 subtype that has infected a chicken is able to spread to other chickens; Prob_Establishment, probability that the H5/H7 LPAI subtype will establish within the flock from one infected chicken; Prob_Subtype_CS, probability that the LPAI H5/H7 subtype established within the flock is able to produce clinical signs within the flock; Proportion_CS, proportion of birds infected with LPAI that will produce clinical signs; Prob_Mutation, probability that LPAI established within the flock will mutate to HPAI; Prob_LPAI_Detection, probability that the farmer will detect and report disease to appropriate officials during LPAI establishment; Prob_HPAI_Detection, probability that HPAI will produce clinical signs with the assumption that the probability of detection is extremely high; Spread_LPAI_Boots, probability that shed-to-shed spread of LPAI will occur via the movement of boots; Spread_LPAI_Equipment, probability that shed-to-shed spread of LPAI will occur via the movement of equipment; Spread_LPAI_Vermin, probability that shed-to-shed spread of LPAI will occur via the movement of vermin such as rats and insects; Spread_LPAI_Aerosol, probability that shed-to-shed spread of LPAI will occur via aerosol; Spread_LPAI_Animals, probability that shed-to-shed spread of LPAI will occur via the movement of other animals including pets; Spread_HPAI_Boots, probability that shed-to-shed spread of HPAI will occur via the movement of boots; Spread_HPAI_Equipment, probability that shed-to-shed spread of HPAI will occur via the movement of equipment; Spread_HPAI_Vermin, probability that shed-to-shed spread of HPAI will occur via the movement of vermin such as rats and insects; Spread_HPAI_Aerosol, probability that shed-to-shed spread of HPAI will occur via aerosol; Spread_HPAI_Animals, probability that shed-to-shed spread of HPAI will occur via the movement of other animals including pets*).

**Figure 2:**
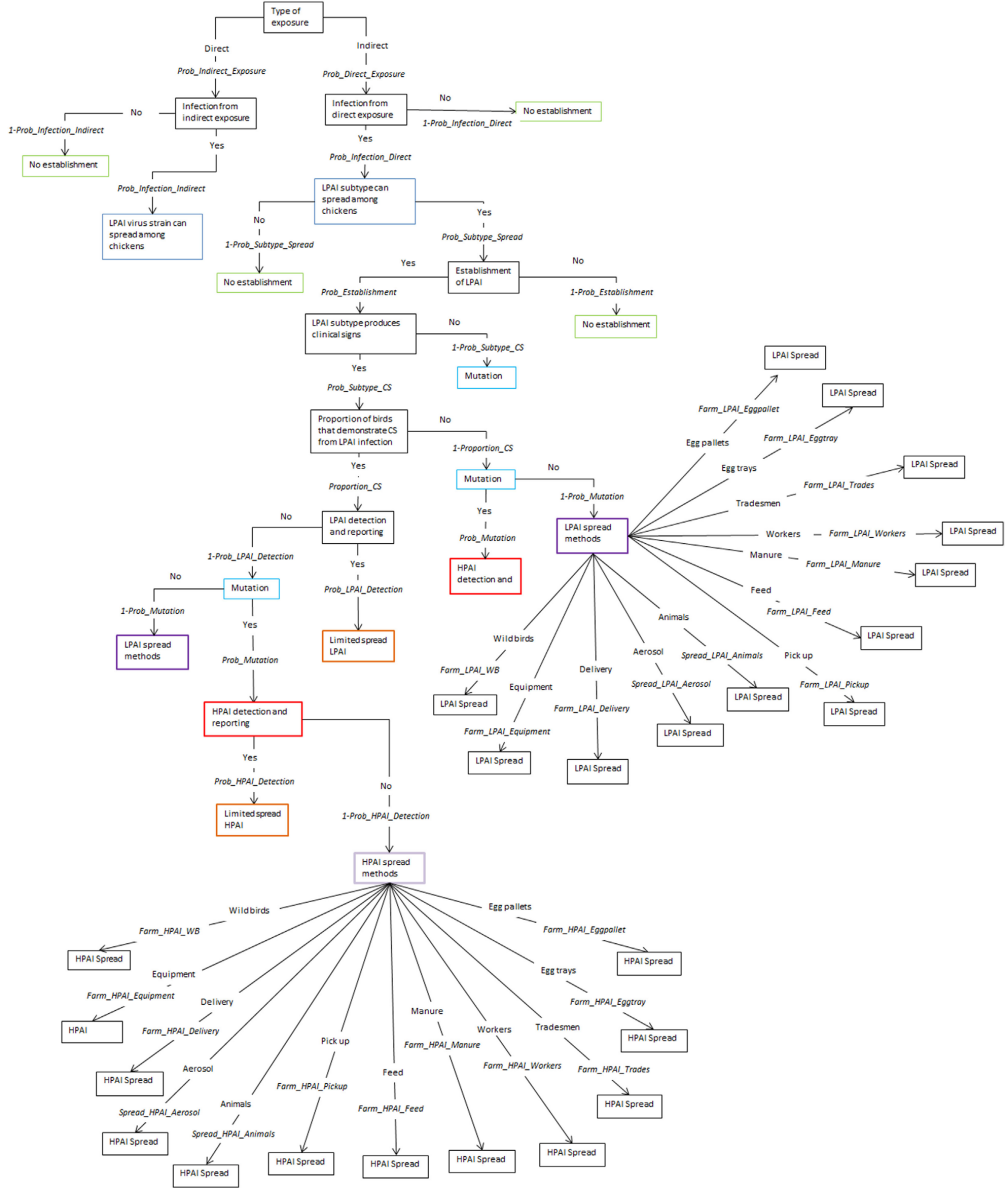
Scenario tree representing the spread pathways of low-pathogenic and high-pathogenic avian influenza (LPAI and HPAI) viruses between farms for Australian commercial layer and meat chicken farms. (*Prob_Indirect_Exposure, probability of indirect exposure of LPAI virus to a commercial chicken; Prob_Direct_Exposure, probability of direct exposure of LPAI virus to a commercial chicken; Prob_Infection_Indirect, probability of infection of LPAI after indirect exposure; Prob_Infection_Direct, probability of infection of LPAI after direct exposure; Prob_Subtype_Spread, probability that the H5/H7 subtype that has infected a chicken is able to spread to other chickens; Prob_Establishment, probability that the H5/H7 LPAI subtype will establish within the flock from one infected chicken; Prob_Subtype_CS, probability that the LPAI H5/H7 subtype established within the flock is able to produce clinical signs within the flock; Proportion_CS, proportion of birds infected with LPAI that will produce clinical signs; Prob_Mutation, probability that LPAI established within the flock will mutate to HPAI; Prob_LPAI_Detection, probability that the farmer will detect and report disease to appropriate officials during LPAI establishment; Prob_HPAI_Detection, probability that HPAI will produce clinical signs with the assumption that the probability of detection is extremely high; Farm_LPAI_Equipment, probability that farm-to-farm spread of LPAI will occur via the movement of equipment; Farm_LPAI_Aerosol, probability that farm-to-farm spread of LPAI will occur via aerosol; Farm_LPAI_Animals, probability that farm-to-farm spread of LPAI will occur via the movement of animals including both farm cats and dogs and vermin; Farm_LPAI_WB, probability that farm-to-farm spread of LPAI will occur via the movement of wild birds; Farm_LPAI_Delivery, probability that farm-to-farm spread of LPAI will occur via the movement of bird delivery transport vehicles; Farm_LPAI_Pickup, probability that farm-to-farm spread of LPAI will occur via the movement of dead and live bird pick up vehicles; Farm_LPAI_Feed, probability that farm-to-farm spread of LPAI will occur via the movement of feed delivery vehicles; Farm_LPAI_Manure, probability that farm-to-farm spread of LPAI will occur via the movement of manure collection systems; Farm_LPAI_Workers, probability that farm-to-farm spread of LPAI will occur via the movement of farm workers; Farm_LPAI_Tradesmen, probability that farm-to-farm spread of LPAI will occur via the movement of tradesmen such as plumbers and electricians; Farm_LPAI_Eggtray, probability that farm-to-farm spread of LPAI will occur via the movement of egg trays; Farm_LPAI_Eggpallet, probability that farm-to-farm spread of LPAI will occur via the movement of egg pallets; Farm_HPAI_Equipment, probability that farm-to-farm spread of HPAI will occur via the movement of equipment; Farm_HPAI_Aerosol, probability that farm-to-farm spread of HPAI will occur via aerosol; Farm_HPAI_Animals, probability that farm-to-farm spread of HPAI will occur via the movement of animals including both farm cats and dogs and vermin; Farm_HPAI_WB, probability that farm-to-farm spread of HPAI will occur via the movement of wild birds; Farm_HPAI_Delivery, probability that farm-to-farm spread of HPAI will occur via the movement of bird delivery transport vehicles; Farm_HPAI_Pickup, probability that farm-to-farm spread of HPAI will occur via the movement of dead and live bird pick up vehicles; Farm_HPAI_Feed, probability that farm-to-farm spread of HPAI will occur via the movement of feed delivery vehicles; Farm_HPAI_Manure, probability that farm-to-farm spread of HPAI will occur via the movement of manure collection systems; Farm_HPAI_Workers, probability that farm-to-farm spread of HPAI will occur via the movement of farm workers; Farm_HPAI_Tradesmen, probability that farm-to-farm spread of HPAI will occur via the movement of tradesmen such as plumbers and electricians; Farm_HPAI_Eggtray, probability that farm-to-farm spread of HPAI will occur via the movement of egg trays; Farm_HPAI_Eggpallet, probability that farm-to-farm spread of HPAI will occur via the movement of egg pallets*).

The spread models used to estimate shed-to-shed and farm-to-farm spread are two separate models and are independent of each other. The same input parameters are used in both models with the exception of the last node that considers the different pathways of spread, shed-to-shed and farm-to-farm. The five pathways for spread between sheds are shown in Figure 1 and the 12 pathways for spread between farms are shown in Figure 2, following the nodes “LPAI spread methods” and “HPAI spread methods”. The input parameters used are described in Table 1 and a detailed description of the nodes is provided in the Supplementary Material. The majority of nodes apply to both LPAI and HPAI spread, with some specific to LPAI or HPAI spread only. The specific nodes for LPAI spread are LPAI spread methods shed-to-shed and LPAI spread methods farm-to-farm. The specific nodes for HPAI spread are HPAI clinical signs, detection, and reporting, HPAI spread methods shed-to-shed, and HPAI spread methods farm-to-farm. The probabilities of the different spread pathways were complementary to each other in the spread scenario tree models (e.g., the sum of the probabilities of all pathways occurring equaled one).

**Table 1:** Nodes, parameter estimates, and input values used for the partial consequence assessment estimating the probability of spread of Avian Influenza (AI) viruses from flocks on both layer and meat commercial chicken farms in Australia^a^.

Node	Branch of node	Parameter estimates	Input values	Data sources
**Parameters that apply to both LPAI and HPAI spread**				
1. Type of exposure	Direct Indirect	Probability that exposure to the virus is direct or indirect exposure based on results from the exposure scenario tree (*Prob_Direct_Exposure; Prob_Indirect_Exposure*)	Prob_Direct_Exposure Average of all direct exposure outputs from the three seasons of the respective farm type^a^ exposure scenario trees. The following values (median; 5–95%) of *Prob_Direct_Exposure* for each farm type were: Non-free range meat chicken (0.24; 0.095–0.47) Free range meat chicken (0.52; 0.28–0.76) Cage layer (0.36; 0.14–0.60) Barn layer (0.32; 0.10–0.59) Free range layer (0.77; 0.60–0.86) *Prob_Indirect_Exposure* *1. Prob_Direct_Exposure*	Exposure section of this study (1)

2. Infection from direct exposure	Yes No	Probability of infection from direct exposure to AI virus in one chicken (*Prob_Infection_Direct*) Average of (probability of infection from intranasal inoculation + probability of infection from gastrointestinal inoculation + probability of infection as a direct in-contact animal)	*Probability of infection from intranasal inoculation* (PrIntranasal) Average LPAI H5N2 viral titers in tracheal swabs of Mallard ducks was 10^3.8^ EID 50/ml over 6 days post inoculation 26/26 chickens inoculated via intranasal route with 10^4.69^ TCID 50/ml H9N2 LPAI became infected 16/18 chickens inoculated via intranasal route with 10^3.69^ TCID 50/ml H9N2 LPAI became infected Therefore, 42 (s) of 46 (n) chickens become infected when inoculated via intranasal route with virus concentration similar to what is naturally excreted from upper respiratory tract from ducks PrIntranasal = Beta (s + 1, n − s + 1) *Probability of infection from gastrointestinal inoculation* (PrGIT) Average LPAI H5N2 viral titers in cloacal swabs of Mallard ducks was 10^2.04^ EID 50/ml over 5 days post inoculation 1/22 chickens inoculated via gastrointestinal route with 10^2.69^ TCID50/ml H9N2 LPAI became infected In natural setting viral titers in duck feces will range considerably, therefore pert distribution used PrGIT = Pert (0, 1/22, 1) *Probability of infection as a direct in-contact animal* (PrContact) 2 in-contact chickens placed directly in-contact with H5N2 LPAI infected chickens (n), 2 became infected (s) PrContact = Beta (s + 1, n − s + 1) *Prob_Infection_Direct* = average (PrIntranasal; PrGIT; PrContact)	Yao et al. (3), Selleck (23), Webster et al. (24)

3. Infection from indirect exposure	Yes No	Probability of infection from indirect exposure to AI virus in one chicken (*Prob_Infection_Indirect*) (Relative likelihood of aerosol exposure × Probability of infection from aerosol + Relative likelihood of all other indirect exposure × Probability of infection from diluted gastrointestinal inoculation)	Relative proportions of the following are taken by summing the two values and dividing each value by the sum: Average of all indirect exposure outputs via aerosol from the three seasons of the respective farm type^a^ exposure scenario tree (PropAerosol) Average of all other indirect exposure outputs from the three seasons of the respective farm type^a^ exposure scenario tree (PropIndirect) *Probability of infection from aerosol* (PrAerosol) Assume virus concentration in air in realistic scenarios is very low from wild birds 0 (s) of 10 (n) chickens exposed to aerosol virus concentration of 10^2.69^ TCID50/ml H9N2 LPAI became infected PrAerosol = Beta (s + 1, n − s + 1) *Probability of infection from gastrointestinal inoculation* (PrGIT) 1/22 chickens inoculated via gastrointestinal route with 10^2.69^ TCID 50/ml H9N2 LPAI became infected 0/31 chickens inoculated via gastrointestinal route with 10^1.69^ TCID 50/ml H9N2 LPAI became infected Therefore, 1 (s) of 53 (n) chickens become infected when inoculated via gastrointestinal route with diluted virus concentration PrGIT = Beta (s + 1, n − s + 1) *Prob_Infection_Indirect* = (PropAerosol × PrAerosol) + (PropIndirect × PrGIT)	Exposure section on this study (1), Yao et al. (3), Jonges et al. (25)

4. Low-pathogenic avian influenza (LPAI) subtype can spread among chickens	Yes No	Probability that the H5/H7 subtype is a particular subtype that can spread among chickens once infected in an individual chicken (*Prob_Subtype_Spread*)	Beta (s + 1, n − s + 1) 18 H5/H7 subtypes exist (n), nine have been recorded as AI outbreaks in chickens across the globe and therefore have the ability to spread (s)	FAO EMPRES-i (4)

5. Establishment of LPAI after infection in one chicken	Yes No	Probability that the virus will establish within the flock after infection in one chicken (*Prob_Establishment*)	Uniform (0.423,0.511) Derived from (1 − Probability of extinction) Probability of extinction of infection calculated with a Poisson branching process using a range of reproduction numbers (R) using real outbreak data	Barnes and Glass (26)

6. LPAI subtype leads to clinical signs in chickens after infection	Yes No	Probability that the LPAI subtype infected within the flock is a subtype that produces clinical signs in chickens (*Prob_Subtype_CS*)	Beta (s + 1, n − s + 1) 52 H5/H7 virus subtypes, some repeated, have been inoculated in chickens (n), 24 caused clinical signs in chickens (s)	Spackman et al. (27), Spickler et al. (2)

7. Proportion of chickens that show clinical signs from LPAI infection	Yes No	Estimated proportion of chickens within a flock that show clinical signs after infected with a LPAI subtype capable of producing clinical signs (*Proportion_CS*)	Beta (s + 1, n − s + 1) 23 chickens were inoculated with LPAI viruses of H5/H7 subtypes (n), 6 showed clinical signs (s)	Mo et al. (28), Jones and Swayne (29)

8. LPAI detection and reporting	Yes No	Probability that the farmer will report clinical signs of LPAI to appropriate officials (*Prob_LPAI_Detection*)	Beta (s + 1, n − s + 1) Non-free range meat chicken farms: 50 answers reported from farmers of unusual signs in chickens (n), 31 answers linked to clinical signs caused by LPAI (s) Free range meat chicken farms: 58 answers reported from farmers of unusual signs in chickens (n), 35 answers linked to clinical signs caused by LPAI (s) Cage layer farms: 27 answers reported from farmers of unusual signs in chickens (n), 19 answers linked to clinical signs caused by LPAI (s) Barn layer farms: 30 answers reported from farmers of unusual signs in chickens (n), 21 answers linked to clinical signs caused by LPAI (s) Free range layer farms: 74 answers reported from farmers of unusual signs in chickens (n), 51 answers linked to clinical signs caused by LPAI (s)	Scott et al. (21), Scott et al. (8), Swayne (30)

9. Mutation of LPAI to high-pathogenic avian influenza (HPAI)	Yes No	Probability that LPAI will mutate to HPAI (*Prob_Mutation*)	Results obtained from expert opinion workshop |10 experts responded using a 4-step elicitation process for all questions The question for this node was: “Imagine 100 sheds each of the following operation types where LPAI has recently been established. In how many of these sheds would LPAI mutate to HPAI?” This question was asked for each farm type. The following values (median; 5–95%) for each farm type (where the sum of the yes and no pathways was 1) were: Non-free range meat chicken (0.068; 0–0.21) Free range meat chicken (0.068; 0–0.20) Cage layer (0.49; 0.065–0.93) Barn layer (0.29; 0.054–0.92) Free range layer (0.29; 0.057–0.92)	Singh et al. (22)

**Parameters that are specific to LPAI spread**				
10. LPAI methods shed to shed	Boots Equipment Vermin Aerosol Pets	Probability that LPAI will spread between sheds via the following pathways: boots, equipment, vermin, aerosol or pets (*Spread_LPAI_Boots; Spread_LPAI_Equipment; Spread_LPAI_Vermin; Spread_LPAI_Aerosol; Spread_LPAI_Animals*)	*Probability of LPAI spread via boots* Beta (s + 1, n − s + 1) of farm answers (PrBoots) Non-free range meat chicken farms: 1/15 (s/n) answers did not use footbaths Free range meat chicken farms: 1/15 (s/n) answers did not use footbaths Cage layer farms: 7/9 (s/n) answers did not use footbaths Barn layer farms: 3/9 (s/n) answers did not use footbaths Free range layer farms: 6/25 (s/n) answers did not use footbaths Probability of virus presence on boots in one day is 1 as survival is longer than one day on this material *Spread_LPAI_Boots* = (PrBoots) × 1 *Probability of LPAI spread via equipment* Beta (s + 1, n − s + 1) of farm answers (PrEquipment) Non-free range meat chicken farms: 6/11 (s/n) answers do not clean equipment between sheds Free range meat chicken farms: 9/9 (s/n) answers do not clean equipment between sheds Cage layer farms: 7/7 (s/n) answers do not clean equipment between sheds Barn layer farms: 6/7 (s/n) answers do not clean equipment between sheds Free range layer farms: 2/23 (s/n) answers do not clean equipment between sheds Probability of virus presence on equipment in one day is 1 as survival is longer than one day on this material *Spread_LPAI_Equipment* = (PrEquipment) × 1 *Probability of LPAI spread via vermin* Beta (s + 1, n − s + 1) of farm answers (PrVermin) Non-free range meat chicken farms: 24/30 (s/n) answers report vermin inside sheds Free range meat chicken farms: 2/30 (s/n) answers report vermin inside sheds Cage layer farms: 17/18 (s/n) answers report vermin inside sheds Barn layer farms: 17/18 (s/n) answers report vermin inside sheds Free range layer farms: 44/50 (s/n) answers report vermin inside sheds Probability of virus presence/survival in vermin (SurvivalVermin): Beta (s + 1, n − s + 1) 0/12 (s/n) LPAI inoculated rats and 73/171 (s/n) LPAI inoculated fly pools were positive on virus isolation *Spread_LPAI_Vermin* = (PrVermin) × (SurvivalVermin) *Probability of LPAI spread via aerosol* Beta (s + 1, n − s + 1) of farm answers (PrAerosol) Non-free range meat chicken farms: 15/15 (s/n) answers had sheds <60 m from each other Free range meat chicken farms: 15/15 (s/n) answers had sheds < 60m from each other Cage layer farms: 9/9 (s/n) answers had sheds <60 m from each other Barn layer farms: 9/9 (s/n) answers had sheds <60 m from each other Free range layer farms: 25/25 (s/n) answers had sheds <60 m from each other Probability of virus presence/survival in air (SurvivalAerosol): Beta (s + 1, n − s + 1) 0/9 (s/n) air samples tested at < 60m from LPAI infected chicken farms were positive for LPAI virus *Spread_LPAI_Aerosol* = (PrAerosol) × (SurvivalAerosol) *Probability of LPAI spread via animals* Beta (s + 1, n − s + 1) of farm answers (PrAnimals) Non-free range meat chicken farms: 0/15 (s/n) answers allow animals inside sheds Free range meat chicken farms: 2/30 (s/n) answers allow animals inside sheds or range areas Cage layer farms: 6/9 (s/n) answers allow animals inside sheds Barn layer farms: 1/9 (s/n) answers allow animals inside sheds Free range layer farms: 13/50 (s/n) answers allow animals inside sheds or range areas Probability of virus presence on other animals in one day is 1 as virus survival is longer than one day on other animals *Spread_LPAI_Animals* = (PrAnimals) × 1	Scott et al. (21); Scott et al. (8); Achenbach and Bowen (31); Nielsen et al. (32); Tiwari et al. (33); Jonges et al. (25); Wood et al. (34)

11. LPAI spread methods farm to farm	Aerosol Infected wild bird Animals (vermin and pets) Bird delivery transport Bird pick up transport (live and dead) Feed delivery transport Manure collection Farm workers Trades people Shared equipment Egg trays^b^ Egg pallets^b^	Probability that LPAI will spread between farms via the following pathways: aerosol, infected wild bird going from one farm to another, other animals including vermin and pets, new bird delivery transport, bird pick up transport both live and dead, feed delivery transport, manure collection, farm workers, trades people such as electricians and plumbers, shared equipment between farms, egg trays^b^, egg pallets^b^ (*Farm_LPAI_Aerosol; Farm_LPAI_WB; Farm_LPAI_Animals; Farm_LPAI_Delivery; Farm_LPAI_Pickup; Farm_LPAI_Feed; Farm_LPAI_Manure; Farm_LPAI_Workers; Farm_LPAI_Trades; Farm_LPAI_Equipment; Farm_LPAI_Eggtray; Farm_LPAI_Eggpallet*)	Results obtained from expert opinion workshop 10 experts responded using a 4-step elicitation process for all questions The question for this node was: “Imagine 100 LPAI established (farm type)^c^ farms. Realistically how many of these will experience LPAI spread to at least one other chicken farm through each of the following pathways?” The values for each pathway and farm type are present in the Supplementary Material	Singh et al. (22)

**Parameters that are specific to HPAI spread**				
12. HPAI clinical signs, detection and reporting	Yes No	Probability that clinical signs will be shown in chickens infected with HPAI and the probability the farmer will detect and report the disease to appropriate officials (*Prob_HPAI_Detection*)	Beta (s + 1, n − s + 1) 52 chickens were inoculated with HPAI viruses of H7 subtypes (n), 52 showed clinical signs (s) Assume extremely high probability farmer will detect clinical signs of HPAI	Selleck (23)

13. HPAI spread methods shed to shed	Boots Equipment Vermin Aerosol Pets	Probability that HPAI will spread between sheds via the following pathways: boots, equipment, vermin, aerosol or pets (*Spread_HPAI_Boots; Spread_HPAI_Equipment; Spread_HPAI_Vermin; Spread_HPAI_Aerosol; Spread_HPAI_Animals)*	*Probability of HPAI spread via boots* Beta (s + 1, n − s + 1) of farm answers (PrBoots) Non-free range meat chicken farms: 1/15 (s/n) answers did not use footbaths Free range meat chicken farms: 1/15 (s/n) answers did not use footbaths Cage layer farms: 7/9 (s/n) answers did not use footbaths Barn layer farms: 3/9 (s/n) answers did not use footbaths Free range layer farms: 6/25 (s/n) answers did not use footbaths Probability of virus presence on boots in one day is 1 as survival is longer than 1 day on this material *Spread_HPAI_Boots* = (PrBoots) × 1 *Probability of HPAI spread via equipment* Beta (s + 1, n − s + 1) of farm answers (PrEquipment) Non-free range meat chicken farms: 6/1 (s/n)1 answers do not clean equipment between sheds Free range meat chicken farms: 9/9 (s/n) answers do not clean equipment between sheds Cage layer farms: 7/7 (s/n) answers do not clean equipment between sheds Barn layer farms: 6/7 (s/n) answers do not clean equipment between sheds Free range layer farms: 2/23 (s/n) answers do not clean equipment between sheds Probability of virus presence on equipment in one day is 1 as survival is longer than one day on this material *Spread_HPAI_Equipment* = (PrEquipment) × 1 *Probability of HPAI spread via vermin* Beta (s + 1, n − s + 1) of farm answers (PrVermin) Non-free range meat chicken farms: 24/30 (s/n) answers report vermin inside sheds Free range meat chicken farms: 2/30 (s/n) answers report vermin inside sheds Cage layer farms: 17/18 (s/n) answers report vermin inside sheds Barn layer farms: 17/18 (s/n) answers report vermin inside sheds Free range layer farms: 44/50 (s/n) answers report vermin inside sheds Probability of virus presence/survival in vermin (SurvivalVermin): Beta (s + 1, n − s + 1) 0/516 (s/n) HPAI exposed rats and 41/59 (s/n) HPAI inoculated flies were positive on virus isolation *Spread_HPAI_Vermin = (PrVermin) × (SurvivalVermin)* *Probability of HPAI spread via aerosol* Beta (s + 1, n − s + 1) of farm answers (PrAerosol) Non-free range meat chicken farms: 15/15 (s/n) answers had sheds <150 m from each other Free range meat chicken farms: 15/15 (s/n) answers had sheds <150 m from each other Cage layer farms: 9/9 (s/n) answers had sheds <150 m from each other Barn layer farms: 9/9 (s/n) answers had sheds <150 m from each other Free range layer farms: 25/25 answers had sheds <150 m from each other Probability of virus presence/survival in air (SurvivalAerosol): Beta (s + 1, n − s + 1) 22/90 (s/n) air samples tested at <60 m from HPAI infected chicken farms were positive for HPAI virus *Spread_HPAI_Aerosol* = (PrAerosol) × (SurvivalAerosol) *Probability of HPAI spread via animals* Beta (s + 1, n − s + 1) of farm answers (PrAnimals) Non-free range meat chicken farms: 0/15 (s/n) answers allow animals inside sheds Free range meat chicken farms: 2/30 (s/n) answers allow animals inside sheds Cage layer farms: 6/9 (s/n) answers allow animals inside sheds Barn layer farms: 1/9 (s/n) answers allow animals inside sheds Free range layer farms: 13/50 (s/n) answers allow animals inside sheds Probability of virus presence on other animals in one day is 1 as virus survival is longer than one day on other animals *Spread_HPAI_Animals* = (PrAnimals) × 1	Scott et al. (21), Scott et al. (8), Tiwari et al. (33), Wood et al. (34), Sawabe et al. (35), Nettles et al. (36), McCluskey (37)

14. HPAI spread methods farm to farm	Aerosol Infected wild bird Animals (vermin and pets) Bird delivery transport Bird pick up transport (live and dead) Feed delivery transport Manure collection Farm workers Trades people Shared equipment Egg trays^b^ Egg pallets^b^	Probability that HPAI will spread between farms via the following pathways: aerosol, infected wild bird going from one farm to another, other animals including vermin and pets, new bird delivery transport, bird pick up transport both live and dead, feed delivery transport, manure collection, farm workers, trades people such as electricians and plumbers, shared equipment between farms, egg trays^b^, egg pallets^b^ (*Farm_HPAI_Aerosol; Farm_HPAI_WB; Farm_HPAI_Animals; Farm_HPAI_Delivery; Farm_HPAI_Pickup; Farm_HPAI_Feed; Farm_HPAI_Manure; Farm_HPAI_Workers; Farm_HPAI_Trades; Farm_HPAI_Equipment; Farm_HPAI_Eggtray; Farm_HPAI_Eggpallet*)	Results obtained from expert opinion workshop 10 experts responded using a four-step elicitation process for all questions The question for this node was: “Imagine 100 HPAI established (farm type)^c^ farms. Realistically how many of these will experience HPAI spread to at least one other chicken farm through each of the following pathways?” The values for each pathway and farm type are present in the Supplementary Material	Singh et al. (22)

*^a^A spread scenario tree was performed for all farm types; non-free range meat chicken, free range meat chicken, cage layer, barn layer and free range layer*.

*^b^These pathways applied to layer farms only; cage layer, barn layer, and free range layer*.

*^c^This question was asked for each farm type*.

### Sensitivity Analysis

The Advanced Sensitivity Analysis on the program @RISK 7.0 (Palisade Corporation, USA) was used to determine the effect of input parameters on the model outputs. The input values varied from 0 to 1 in thirds (0, 0.3, 0.6, 1). Each input value of interest was assessed in a simulation of 1,000 iterations while all other input values were fixed to their base value. The model outputs assessed were the probability of LPAI and HPAI spread between both sheds and farms per farm type.

The effect of the following inputs of LPAI and HPAI spread between sheds and farms were investigated: (1) Probability that the H5/H7 LPAI subtype will establish within the flock from one infected chicken *(Prob_Establishment)*; (2) Probability that LPAI established within the flock will mutate to HPAI *(Prob_Mutation)*; (3) Probability that the farmer will detect and report disease to appropriate officials during LPAI establishment *(Prob_LPAI_Detection)*; (4) Probability that HPAI will produce clinical signs with the assumption that the probability of detection is extremely high *(Prob_HPAI_Detection)*.

In addition, the impact of the probability of spread to another shed or farm through any of the pathways considered in this assessment, which is dependent to a high extent on the level of biosecurity implemented on farm, was also investigated. As the probabilities of the different spread pathways were complementary to each other in the spread scenario tree models, each pathway has the same influence on the probability of spread on the sensitivity analysis. As such, only one pathway probability is included in the sensitivity analysis and the generic term *Prob_PathwaySpread* is used.

## Results

### Probabilities of LPAI and HPAI Spread

**Figure 3:**
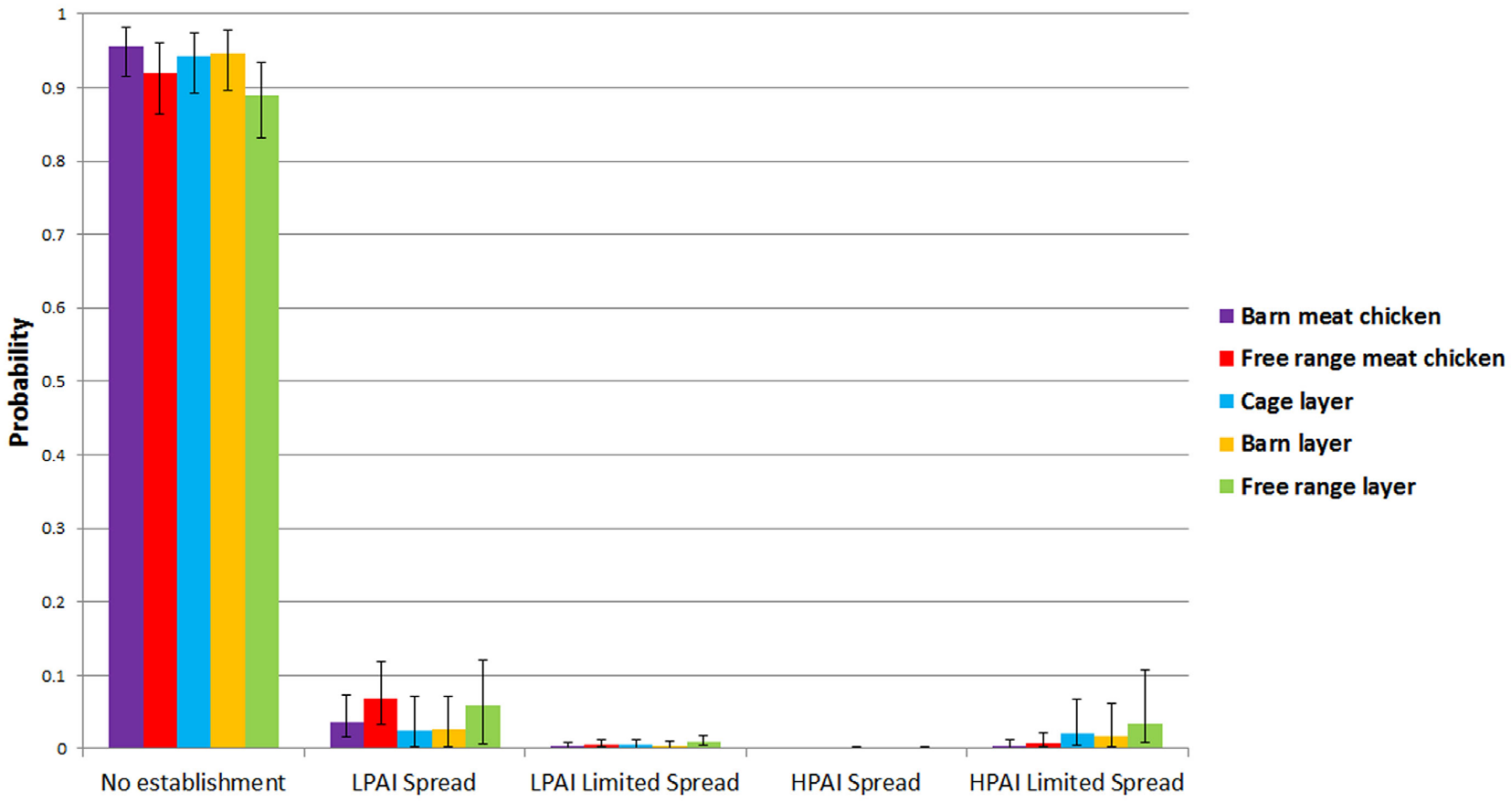
Median (5 and 95 percentiles) probabilities of no establishment and of low-pathogenic avian influenza (LPAI) and high-pathogenic avian influenza (HPAI) spread and limited spread between sheds and farms for the commercial chicken farm types (barn meat chicken, free range meat chicken, cage layer, barn layer, free range layer) after one chicken is exposed to LPAI in Australia.

Results from the spread models provided the overall probabilities of no establishment of LPAI and of LPAI and HPAI limited spread and LPAI and HPAI spread between both sheds and farms, given one chicken is exposed to LPAI virus from one wild bird in Australia at any point of time. The results are summarized in Table 2 and Figure 3. The pathways involved in calculating these probabilities incorporated the probability of LPAI infection in a chicken after exposure and the probability that the virus is able to spread and establish among chickens within a shed. For all farm types, the most likely end-point after one chicken is exposed and infected with LPAI is no establishment. For each pathotype, the overall probabilities of spread are identical for each farm type between sheds and between farms. The results also show that for all farm types, the probability of limited LPAI spread is lower than that of limited HPAI spread; that LPAI spread is more likely to occur than limited LPAI spread; and that HPAI spread is less likely to occur than limited HPAI spread.

**Table 2:** Median (5 and 95 percentiles) probabilities of no establishment and of low-pathogenic avian influenza (LPAI) and high-pathogenic avian influenza (HPAI) spread and limited spread between sheds and farms for the commercial chicken farm types (barn meat chicken, free range meat chicken, cage layer, barn layer, and free range layer) after exposure of one chicken to LPAI from one wild bird in Australia.

Farm type	Median	5%	95%	*F* statistic (degrees of freedom);*P*-value
**No establishment**				
Barn meat chicken	0.96	0.92	0.98	*F*(4,4995) = 990.03; <0.0001
Free range meat chicken	0.92	0.86	0.96	
Cage layer	0.94	0.89	0.97	
Barn layer	0.95	0.9	0.98	
Free range layer	0.89	0.83	0.93	
**Probability of LPAI spread**				
Barn meat chicken	0.037	0.015	0.073	*F*(4,4995) = 490.61; <0.0001
Free range meat chicken	0.068	0.033	0.12	
Cage layer	0.027	0.0031	0.079	
Barn layer	0.026	0.003	0.071	
Free range layer	0.059	0.0071	0.12	
**Probability of HPAI spread**				
Barn meat chicken	2.47 × 10^−5^	0	0.00025	*F*(4,4995) = 164.01; <0.0001
Free range meat chicken	4.60 × 10^−5^	0	0.00043	
Cage layer	0.00022	1.01 × 10^−5^	0.0019	
Barn layer	0.00017	7.33 × 10^−6^	0.0016	
Free range layer	0.00037	1.68 × 10^−5^	0.0031	
**Probability of limited LPAI spread**				
Barn meat chicken	0.0032	0.0011	0.008	*F*(4,4995) = 515.67; <0.0001
Free range meat chicken	0.0058	0.0022	0.013	
Cage layer	0.0048	0.0017	0.012	
Barn layer	0.0044	0.0015	0.011	
Free range layer	0.0092	0.004	0.019	
**Probability of limited HPAI spread**				
Barn meat chicken	0.0044	0.0012	0.013	*F*(4,4995) = 624.38; <0.0001
Free range meat chicken	0.0084	0.0025	0.022	
Cage layer	0.021	0.0044	0.068	
Barn layer	0.016	0.0035	0.063	
Free range layer	0.034	0.0087	0.11	

Low-pathogenic avian influenza and HPAI spread occur when the randomly selected values for the beta distribution for the probability of detection and reporting in the spread model are very low or zero. The probabilities of LPAI spread between sheds and farms, although low for all farms, were estimated to be highest in free range farms compared to other farm types. The model estimated a median probability of LPAI spread of 0.068 and 0.059 for free range meat chicken and layer farms, respectively. Among indoor farms, the probability (median; 5–95%) of LPAI spread between sheds and farms is higher in barn meat chicken farms (0.037; 0.015–0.073) when compared to the indoor layer farm types; cage layer (0.027; 0.0028–0.079) and barn layer (0.026; 0.0030–0.071). The probabilities of HPAI spread between sheds and farms are lower than that of LPAI spread for all farm types (Table 2).

### Probabilities of the Different Spread Pathways

**Figure 4:**
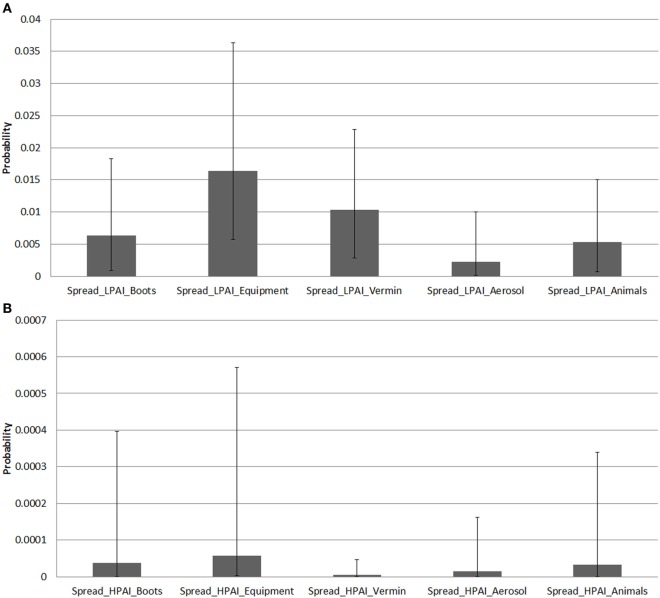

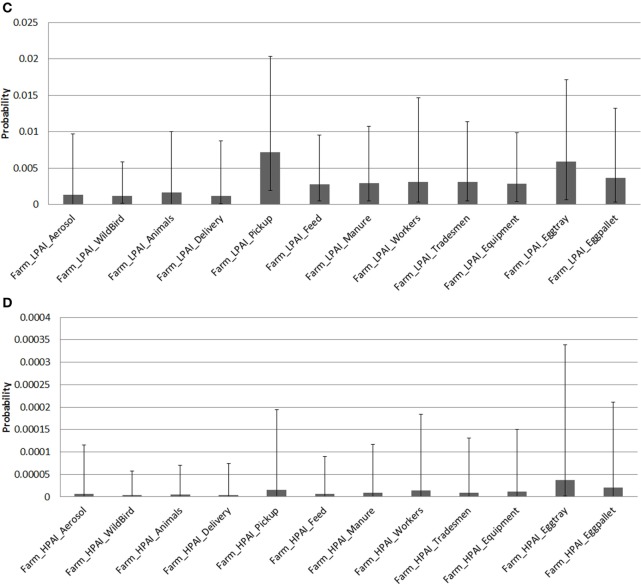
Average median (5 and 95 percentiles) probabilities of low-pathogenic avian influenza (LPAI) and high-pathogenic avian influenza (HPAI) spread pathways between sheds and farms of the commercial chicken farm types (barn meat chicken, free range meat chicken, cage layer, barn layer, free range layer) after one chicken is exposed to LPAI in Australia. **(A)** Average median probabilities of LPAI spread pathways between sheds. *Spread_LPAI_Boots, Probability that shed-to-shed spread of LPAI will occur via the movement of boots; Spread_LPAI_Equipment, Probability that shed-to-shed spread of LPAI will occur via the movement of equipment; Spread_LPAI_Vermin, Probability that shed-to-shed spread of LPAI will occur via the movement of vermin such as rats and insects; Spread_LPAI_Aerosol, Probability that shed-to-shed spread of LPAI will occur via aerosol; Spread_LPAI_Animals, Probability that shed-to-shed spread of LPAI will occur via the movement of other animals including farm cats and dogs*. **(B)** Average median probabilities of HPAI spread pathways between sheds. *Spread_HPAI_Boots, Probability that shed-to-shed spread of HPAI will occur via the movement of boots; Spread_HPAI_Equipment, Probability that shed-to-shed spread of HPAI will occur via the movement of equipment; Spread_HPAI_Vermin, Probability that shed-to-shed spread of HPAI will occur via the movement of vermin such as rats and insects; Spread_HPAI_Aerosol, Probability that shed-to-shed spread of HPAI will occur via aerosol; Spread_HPAI_Animals, Probability that shed-to-shed spread of HPAI will occur via the movement of other animals including farm cats and dogs*. **(C)** Average median probabilities of LPAI spread pathways between farms. *Farm_LPAI_Equipment, Probability that farm-to-farm spread of LPAI will occur via the movement of equipment; Farm_LPAI_Aerosol, Probability that farm-to-farm spread of LPAI will occur via aerosol; Farm_LPAI_Animals, Probability that farm-to-farm spread of LPAI will occur via the movement of animals including both farm cats and dogs and vermin; Farm_LPAI_WB, Probability that farm-to-farm spread of LPAI will occur via the movement of wild birds; Farm_LPAI_Delivery, Probability that farm-to-farm spread of LPAI will occur via the movement of bird delivery transport vehicles; Farm_LPAI_Pickup, Probability that farm-to-farm spread of LPAI will occur via the movement of dead and live bird pick up vehicles; Farm_LPAI_Feed, Probability that farm-to-farm spread of LPAI will occur via the movement of feed delivery vehicles; Farm_LPAI_Manure, Probability that farm-to-farm spread of LPAI will occur via the movement of manure collection systems; Farm_LPAI_Workers, Probability that farm-to-farm spread of LPAI will occur via the movement of farm workers; Farm_LPAI_Tradesmen, Probability that farm-to-farm spread of LPAI will occur via the movement of tradesmen such as plumbers and electricians; Farm_LPAI_Eggtray, Probability that farm-to-farm spread of LPAI will occur via the movement of egg trays; Farm_LPAI_Eggpallet, Probability that farm-to-farm spread of LPAI will occur via the movement of egg pallets*. **(D)** Average median probabilities of HPAI spread pathways between farms. *Farm_HPAI_Equipment, Probability that farm-to-farm spread of HPAI will occur via the movement of equipment; Farm_HPAI_Aerosol, Probability that farm-to-farm spread of HPAI will occur via aerosol; Farm_HPAI_Animals, Probability that farm-to-farm spread of HPAI will occur via the movement of animals including both farm cats and dogs and vermin; Farm_HPAI_WB, Probability that farm-to-farm spread of HPAI will occur via the movement of wild birds; Farm_HPAI_Delivery, Probability that farm-to-farm spread of HPAI will occur via the movement of bird delivery transport vehicles; Farm_HPAI_Pickup, Probability that farm-to-farm spread of HPAI will occur via the movement of dead and live bird pick up vehicles; Farm_HPAI_Feed, Probability that farm-to-farm spread of HPAI will occur via the movement of feed delivery vehicles; Farm_HPAI_Manure, Probability that farm-to-farm spread of HPAI will occur via the movement of manure collection systems; Farm_HPAI_Workers, Probability that farm-to-farm spread of HPAI will occur via the movement of farm workers; Farm_HPAI_Tradesmen, Probability that farm-to-farm spread of HPAI will occur via the movement of tradesmen such as plumbers and electricians; Farm_HPAI_Eggtray, Probability that farm-to-farm spread of HPAI will occur via the movement of egg trays; Farm_HPAI_Eggpallet, Probability that farm-to-farm spread of HPAI will occur via the movement of egg pallets*.

Results of the probability of LPAI and HPAI spread between sheds and farms are summarized in Figure 4, which presents the averages of the median, 5% and 95% probability values per pathway among all farm types and provides a comparison of relative probability of spread between pathways that does not explicitly consider the volume and frequency of each respective pathway occurring.

The pathways of spread between sheds were estimated using farm survey data to determine the proportion of farms that would perform or have specific practices or pathways for each farm type. This was combined with scientific literature to determine the survival of the virus on each of these pathways, and similar volume and frequency for each pathway were assumed. The pathway of LPAI spread between sheds (Figure 4A) with the highest average median probability was spread via equipment (0.015; 0.0058–0.036), followed by vermin (0.010; 0.0028–0.023) and then boots (0.0064; 0.00087–0.018). When the results of each farm type were assessed, the pathway of spread via equipment was the pathway with the highest median probability of LPAI spread between sheds for each farm type except free range layer farms. For this farm type, the pathway of LPAI spread between sheds with the highest median probability was spread via vermin (0.019; 0.0022–0.041).

The pathway of HPAI spread between sheds (Figure 4B) with the highest average median probability was also spread via equipment (5.76 × 10^−5^; 1.90 × 10^−6^–0.00057). All farm types except free range layer farms had the pathway of spread via equipment as the pathway with the highest median probability of HPAI spread between sheds. For free range farms, the pathway with the highest median probability was spread via animals (8.93 × 10^−5^; 2.57 × 10^−6^–0.001) (data not shown in Figure 4).

The pathways of spread between farms were estimated from expert opinion which is assumed to have considered variation in volume and frequency and virus survival between pathways. The pathway of LPAI spread between farms (Figure 4C) with the highest average median probability was spread via bird pick up systems (0.0072; 0.0019–0.02), followed by egg trays (0.0059; 0.00066–0.017). The latter applies to only layer farm types. When assessing each farm type on its own, the pathway with the highest median probability of LPAI spread between farms was bird pick up systems for both barn and free range meat chicken farm types. Spread via egg trays was the pathway with the highest median probability of LPAI spread between farms for all layer farms.

The pathway of HPAI spread between farms (Figure 4D) with the highest average median probability was spread via egg trays (3.70 × 10^−5^; 1.47 × 10^−6^–0.00034), followed by egg pallets (2.07 × 10^−5^; 7.86 × 10^−7^–0.00021), bird pick up systems (1.57 × 10^−5^; 4.83 × 10^−7^–0.00019), and farm workers (1.41 × 10^−5^; 4.43 × 10^−7^–0.00018). The former two apply to layer farms only. For individual farm types, and similar to that for LPAI, the pathway of HPAI spread between farms with the highest median probability was bird pick up systems for barn and free-range meat chicken farm types. Spread via egg trays was the pathway with the highest median probability of HPAI spread between farms for all layer farms.

### Spread Sensitivity Analysis

**Figure 5:**
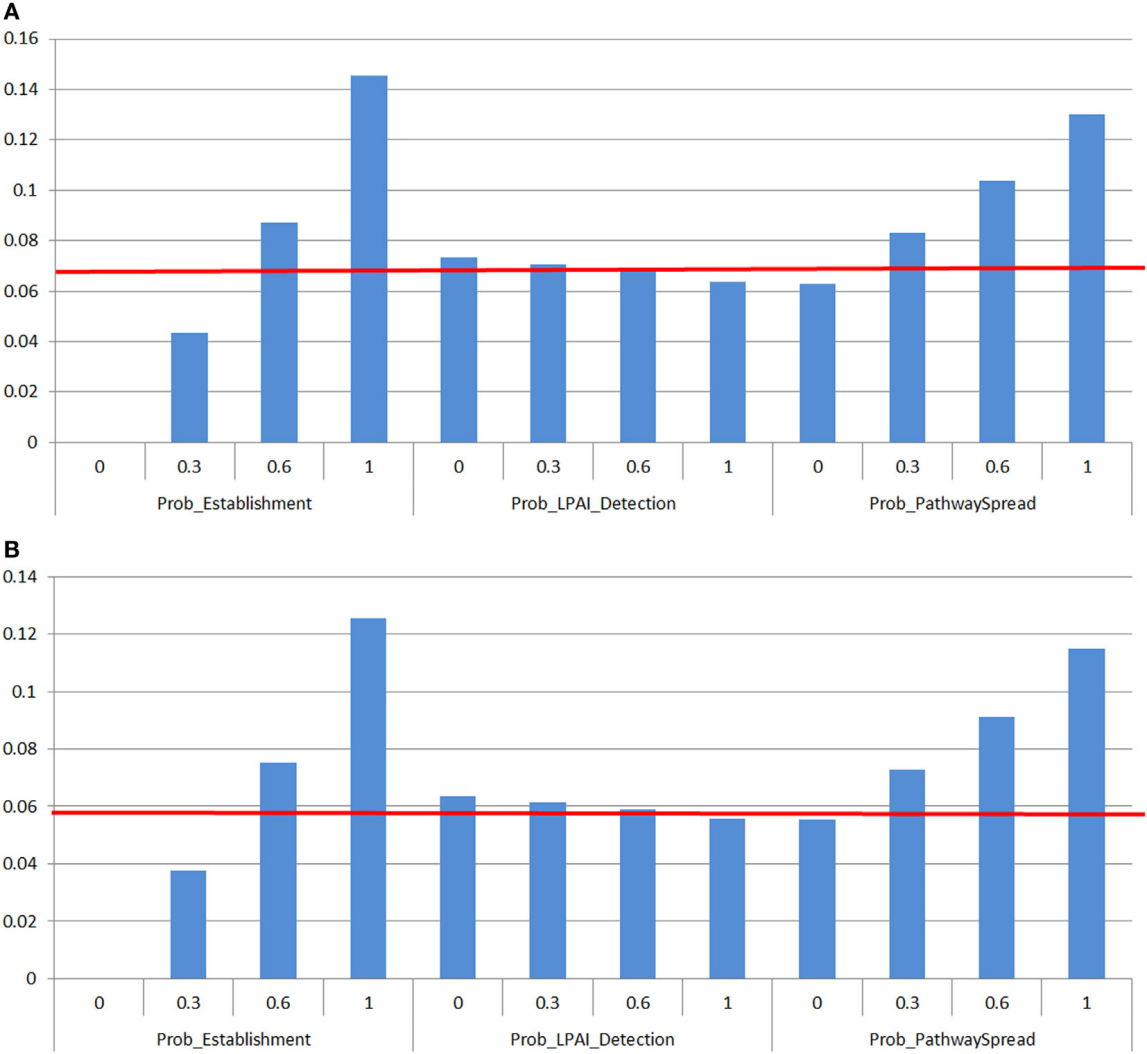

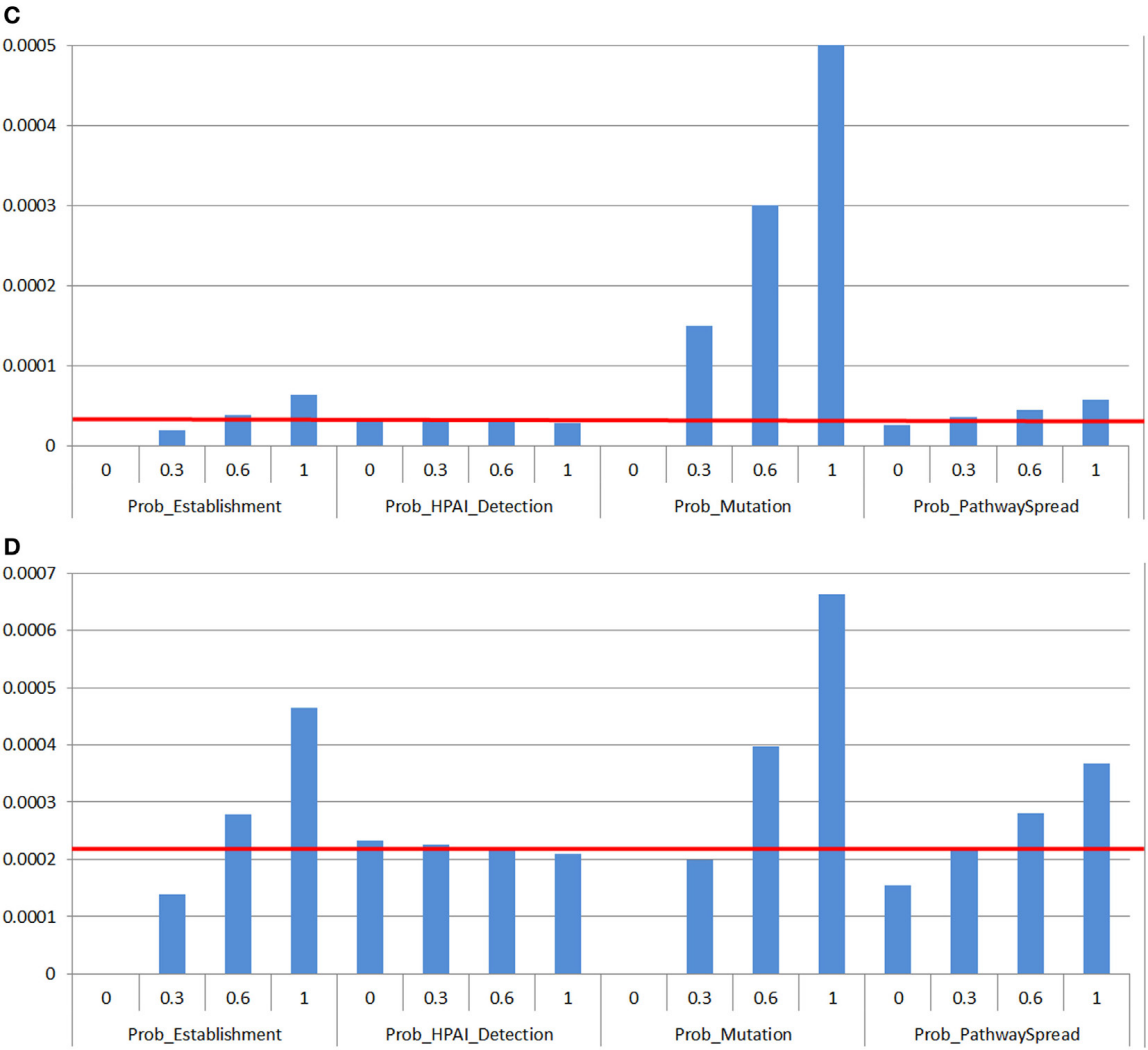
Results of the sensitivity analysis on the spread assessment depicting the change in probability (Y-axis) on the median overall probability of low-pathogenic avian influenza (LPAI) or high-pathogenic avian influenza (HPAI) spread (horizontal line) between sheds on a commercial poultry farm and between commercial poultry farms after exposure of one chicken to low-pathogenic avian influenza (LPAI) virus from wild birds in Australia with changes of certain input variables listed in Table 1 (*X*-axis). Results were obtained from a simulation of 1,000 iterations using @Risk’s Advanced Sensitivity Analysis. The outcomes were similar in proportional increase in value among all farm types so only one example of a meat chicken or layer farm type per LPAI **(A,B)** or HPAI **(C,D)** spread between sheds and farms was used. **(A)** Sensitivity analysis on input parameters affecting the probability of LPAI spread between sheds and farms on free range meat chicken farm types. **(B)** Sensitivity analysis on input parameters affecting the probability of LPAI spread between sheds and farms on free range layer farm types. **(C)** Sensitivity analysis on input parameters affecting the probability of HPAI spread between sheds and farms on barn meat chicken farm types. **(D)** Sensitivity analysis on input parameters affecting the probability of HPAI spread between sheds and farms on cage layer farm types. *Prob_Establishment, Probability that the H5/H7 LPAI subtype will establish within the flock from one infected chicken; Prob_Mutation, Probability that LPAI established within the flock will mutate to HPAI; Prob_LPAI_Detection, Probability that the farmer will detect and report disease to appropriate officials during LPAI establishment; Prob_HPAI_Detection, Probability that HPAI will produce clinical signs with the assumption that the probability of detection is extremely high; Prob_PathwaySpread, Probability of any one of the spread pathways identified, with consideration of the complementary changes for all other spread pathways that will result given sum of the probabilities of all pathways occurring equaled one*.

Figure 5 shows the outputs of the spread sensitivity analysis, which depicts an example of one meat chicken or layer farm type per LPAI (Figures 5A,B) or HPAI (Figures 5C,D) spread between sheds and farms, as the sensitivity analysis outcomes were similar in proportional increase in value among all farm types. In addition, no difference on the spread sensitivity analyses for spread between sheds and spread between farms was observed.

According to the spread sensitivity analysis, the most influential parameter for LPAI spread between sheds and farms was the probability of establishment (Figures 5A,B). When the probability of establishment is increased to 100% (base value 0.47 for all farm types), there is an approximate 2.1 to 2.2-fold increase on the probability of LPAI spread between sheds and farms for all farm types.

The probability of mutation was the most influential parameter affecting the probability of HPAI spread between sheds and farms for all farm types. When this probability is increased to 100% (base value 0.070, 0.070, 0.50, 0.28, 0.30 for barn meat chicken, free range meat chicken, cage layer, barn layer and free range layer farms, respectively), there is at least a 3.5-fold increase on the probability of HPAI spread between both sheds and farms for all farm types (Figures 5C,D). The influence of the probability of mutation is most substantial on meat chicken farm types where there is an approximate 17-fold increase on the probability of HPAI spread between both sheds and farms within these farm types. The next most influential parameter on HPAI spread between sheds and farms was the probability of establishment where results obtained were similar to those seen with the LPAI spread sensitivity analysis described above.

The impact of the probability of detection on spread of LPAI and HPAI does not seem to be very significant. When this probability is increased to 100%, there is only an approximate 0.05-fold decrease on the probability of both LPAI (base value between 0.60 and 0.70 for all farm types) and HPAI (base value 0.99 for all farm types) spread between sheds and farms for all farm types.

Investigation of the spread pathways revealed that when the probability of any of these pathways is increased to 100% (base values ranging from 0.00034 to 0.040 and 3.87 × 10^−7^ and 8.83 × 10^−5^ for LPAI and HPAI spread, respectively), there is an approximate 1.5 to 2-fold increase on the probability of LPAI and HPAI spread between sheds and between farms for all farm types. This enabled evaluation of the change in probability of spread with implementation or presence of biosecurity practices that act on these spread pathways.

## Discussion

### The Probability of Spread

The most likely pathway or outcome after one chicken is exposed and infected with LPAI is no establishment of the infection. This is supported by East et al. (38) where in all 17 samples tested positive for AI antibodies in the sentinel free-range flocks, there was no evidence of chicken-to-chicken transmission. However, these results contrast with work performed at the Australian Animal Health Laboratory (AAHL) where chickens inoculated and subsequently infected with various LPAI subtypes were placed in direct contact with other chickens. All chickens in direct contact with these infected chickens subsequently became infected (23). In addition, the spread model assumes only one chicken is exposed to the virus; it is unknown how many chickens are exposed to the virus and over what time period in an Australian context. Therefore, in order for model validation to occur, sampling of commercial chickens to determine their level of exposure to LPAI must be performed.

The overall probabilities of spread are identical for shed-to-shed and farm-to-farm spread for each farm type and pathotype (presented in Table 2), and this is due to the only difference being the specific pathways of spread which are represented in the last node of the scenario tree (Figures 1 and 2). The probabilities of LPAI spread between sheds and farms are highest in free range farms. As previously mentioned, the spread model incorporates the probability of LPAI infection after the first bird has been exposed, where this probability is higher after direct exposure compared to indirect exposure. As such, the higher probability of LPAI spread in free range farms is due to exposure of the exposed bird on these farms to more likely be via direct pathways. Among non-free-range farms, the probability of LPAI spread, although similar, is slightly higher in barn meat chicken farms compared to the indoor layer farm types, due to the higher threshold of detection and reporting of sick and dead chickens in meat chicken farms compared to layer farms. The higher threshold provides more opportunity for the virus to spread before it is detected. In contrast, the probability of HPAI spread in meat chicken farms is lower than that of layer farms due to the short-lived nature of meat chicken birds leading to a lower probability of mutation in meat chicken birds compared to layer birds. This is reflected in expert opinion answers which informed the mutation parameter and gave a higher probability of mutation for layer farms compared to meat chicken farms (22).

Relative comparisons of these results to other countries can only be made for countries with similar LPAI and HPAI situations such as Australia, i.e., countries in which LPAI and HPAI are not endemic in poultry and HPAI is not endemic in wild birds. Countries in which HPAI H5N1 is endemic in poultry include Bangladesh, China, Egypt, India, Indonesia, and Vietnam (39). Similarly, comparisons should only be made to those countries that have effective protocols setup to deal with positive detections to limit spread. In Australia, this is written in the Australian Veterinary Emergency Plan (AUSVETPLAN) for avian influenza, which was developed and agreed upon by government and the poultry industry. In the United Kingdom (UK) and United States of America (USA), similar protocols are written in the Notifiable Avian Influenza Disease Control Strategy and HPAI Response Plan (The Red Book), respectively (40–42). The UK experienced 11 HPAI outbreaks since 2006, all of which were effectively eradicated by destroying all birds on infected premises, comparable to the results of this study which indicate limited HPAI spread to occur more often than HPAI spread (43). However, the USA has experienced more extensive HPAI outbreaks involving dozens of farms, which cost over hundreds of millions of dollars to effectively eradicate. These include the HPAI outbreaks that occurred in Pennsylvania in 1983 and 1984, and the more recent HPAI outbreaks since 2014 that affected more than 10 USA states (44, 45). Suggested factors influencing these extensive HPAI outbreaks in the USA include poor biosecurity between farms, and high levels of exposure to AI virus in poultry farms in general, leading to numerous separate introduction and infection events in addition to spread between sheds and between farms (37).

### The Probability of Spread and the Probability of Limited Spread

The spread models revealed that for all farm types, the probability of LPAI spread is greater than the probability of limited LPAI spread. This is because detection and reporting is less likely to occur following LPAI establishment and so control measures are less likely to put into place that will limit LPAI spread. In contrast, the spread models indicate that limited HPAI spread is more likely to occur than HPAI spread due to the high probability farmers will detect and report the changes in morbidity and mortality that follow HPAI establishment in a chicken flock. In general, there is limited information to determine if shed-to-shed spread has occurred on Australian LPAI-infected farms. There is evidence that shed-to-shed spread may have occurred on two farms; specifically chickens in several sheds on one farm were seropositive to LPAI H6N2 in 2006 and LPAI H9N2 was detected in three sheds on a turkey farm in 2012 (46). However, it is also possible that independent introductions and infections occurred on the sheds of these farms instead of spread between sheds. There has only been one incursion to date with evidence of farm-to-farm LPAI spread in Australia; investigation of the 2012 H9N2 incursion identified a second infected turkey farm during trace back surveillance from the first turkey farm. This second turkey farm showed no clinical signs or increased mortality (14). As mentioned, it is very likely that LPAI detections in Australia are underreported due to these being non-clinical LPAI infections which provides credibility to the outputs of the spread model; that the probability of LPAI spread is greater than that of limited LPAI spread.

Most farms in Australia in which HPAI occurred had the virus spread to other sheds within the farm. However, all outbreaks were effectively controlled via the stamping out procedure and resulting in limited farm-to-farm spread (12, 47, 48). It is likely that the outputs of the spread model which indicate that the probability of HPAI spread is lower than that of limited HPAI spread reflect what has been experienced in Australia; this is easily seen with the farm-to-farm spread model.

### The Different Pathways of Spread

The different pathways of LPAI and HPAI spread between sheds have differing probabilities. For LPAI spread between sheds, equipment and vermin were the most likely pathways and aerosol was the least likely pathway. For HPAI spread between sheds, equipment and boots were the most likely pathways and vermin was the least likely pathway. This is largely due to differences in the survival or detection of the virus reported in the literature relevant to these different pathways. LPAI spread via aerosol is regarded as an unlikely pathway in the literature, but detections of HPAI in air samples have been relatively frequently reported, particularly during the 2015 HPAI outbreaks in USA (25, 37). This is likely due to the higher levels of viral replication that occurs in the respiratory tract of birds with HPAI infection compared to LPAI infection (5). The relatively low probability of HPAI spread between sheds via vermin estimated in this study is likely due to how the input parameters in relation to this pathway were calculated. The input parameters were based on several studies where no virus isolation was obtained after exposure of vermin to AI viruses. It is generally been concluded that mice and rats do not play significant roles in the spread of AI virus but insects may (31, 32). In a study where a large number (n = 516) of samples were taken from rodents exposed to HPAI, no positive virus isolations were obtained (36). Similarly, a study where 12 rodents were inoculated with LPAI, no positive virus isolations were identified (31). The feeding of flies with LPAI and HPAI resulted in lower proportions of positive virus isolations from flies fed HPAI compared to LPAI (32, 35). The pathway of shed-to-shed spread via vermin is possibly more significant for LPAI than HPAI.

When considering the results of this study, it must be remembered that the volume and frequency of the different spread pathways between both sheds and farms were not explicitly incorporated in the spread model. For shed-to-shed spread, these pathways were estimated using farm survey data in combination with scientific literature. The farm survey data were used to determine the proportion of farms that would perform or have specific practices or pathways for each farm type and scientific literature was used to determine the probability of survival of the virus on these pathways. It is known that there is a high frequency of daily movements between sheds and if incorporated in the model, may indicate that HPAI spread between sheds is more likely than limited HPAI spread which would actually explain the high incidence of HPAI spread between sheds on farms affected by HPAI outbreaks in Australia (12, 47, 48). This contrasts with the farm-to-farm spread pathways which were informed by expert opinion due to the lack of information in relation to these pathways. Expert understanding and answers of parameters influencing spread by each pathway can be assumed to have included consideration of the volume and frequency of occurrence and the survival of the virus for each pathway.

The output probabilities from the farm-to-farm spread model on the differing pathways of spread largely reflect the expert opinion answers where relatively higher probabilities of farm-to-farm spread were given to pickup trucks, egg trays, and egg pallets. These comparisons can be made from the model output results in Table 2 and the values in the Supplementary Material derived from expert opinion that were used to inform the pathways between farms (22). Expert estimates were largely influenced by the previous Australian HPAI outbreaks. An epidemiological investigation of the 2013 HPAI outbreak in Young, NSW suggested that the most likely route of spread of this virus to another farm was the contamination of cardboard egg trays (18). Similarly, a dead bird pick-up vehicle which visited multiple farms was the only identifiable link between farms that were affected by the 1997 HPAI outbreak in Tamworth, NSW (17). This compares with an expert opinion elicitation workshop published in 2011, which estimated the probability of HPAI spread between poultry farms to inform models simulating the transmission and control of HPAI epidemics in the Australian poultry industries. The results of this workshop showed that meat chicken pick up crews followed by slaughter crews, manure collection, and cardboard egg trays were rated as the most likely probabilities of transmission between farms (49). Differences observed between the two expert elicitations could be due to the time difference, as the 2012 and 2013 HPAI outbreaks had not yet occurred when the first expert elicitation was conducted.

### Spread Sensitivity Analysis

There were no differences in values or trends on the spread sensitivity analyses of spread between sheds and spread between farms due to the identical structures of the models as described previously. The analyses revealed that the probability of establishment was an important influential parameter on the probability of LPAI and HPAI spread, as well as the probability of mutation on HPAI spread. Although influential, these parameters depend on virus properties and as such cannot be changed by human intervention, and there are large uncertainties associated with these mechanisms (50). Mutation from LPAI to HPAI has particularly large unknowns regarding its probability. A recent review of 42 HPAI outbreaks from 1959 to 2016, most of which involved chickens and turkeys as the initial species, concluded that emergence of HPAI can vary from a few days to a couple of years. It also considered that factors such as poultry age, size of the index farm, and type of farm management do not appear to contribute significantly to HPAI emergence (51). The expert opinion workshop also demonstrated very different estimated probabilities for mutation among the experts (22). The variation of R, which was used to estimate the probability of establishment in the current study, is significant in previous literature, even within the same pathotype and subtype (49, 52, 53). As there is insufficient knowledge about mutation at present to in any way alter the likelihood of its occurrence, the control of LPAI and HPAI spread is therefore mainly reliant on on-farm biosecurity actions.

The detection and reporting parameters were found not to be a significantly influential parameter on the probability of LPAI and HPAI spread. This is supported by modeling work of Barnes and Glass (26), which demonstrated a high probability that a second shed is already infected with HPAI by the time initial infection is detected and reported using typical daily and weekly mortality rates for all farm types. In addition, the index formula described above used to calculate the number of days from infection in the first chicken to establishment, detection and reporting of LPAI also supports the small influence of detection, and reporting on the overall probability of spread. This formula revealed a long time period of at least 70 days for all farm types; within this time period, it is very possible that spread has already occurred to other sheds or farms due to the high level of movements between sheds and farms on all farm types (54, 55). This compares with previous modeling studies which revealed the high significance of detection and reporting in limiting spread of an AI outbreak. However, these studies assessed different but related factors to detection and reporting; including the impact of changing the detection threshold, performing frequent sampling of farms considered high risk, and ensuring prompt action after detection. In contrast, this study assumed a relatively fixed detection threshold based on farmer answers on unusual clinical signs, and therefore the changing parameter in the sensitivity analysis is simply a change in the proportion of farms that will detect and report at this relatively fixed detection threshold. Considerations to further evaluate the significance of detection and reporting are therefore described below.

The spread pathways on the scenario tree models were complementary to each other where the sum of all LPAI or HPAI spread pathway probabilities of one scenario tree model was one. Therefore, the spread sensitivity analysis could not accurately portray the effects of changing one spread pathway as this would result in complementary changes to the other spread pathways; each spread pathway had the same influence on the probability of spread. This was depicted as “*Prob_PathwaySpread*” which represented changing the probability of any one spread pathway and the complementary changes to the probabilities of the other spread pathways. Increasing any spread pathway to 100%, which results in 0% probability of all other spread pathways, resulted in approximate doubling of the overall median probability of either LPAI or HPAI spread. This means if the probability of any pathway is certain to occur, and all other pathways are certain not to occur, the probability of either LPAI or HPAI spread is approximately doubled. In reality all other spread pathways will have a probability greater than zero of occurring. It is therefore expected that in a model where such pathways are not complementary to each other, the cumulative effect of increasing the probabilities of each individual pathway will result in greater than doubling the overall probability of LPAI or HPAI spread. The spread pathways are therefore significant influential parameters on the overall probability of spread and are dependent on biosecurity on the farm. Other highly influential parameters in the spread model such as the probability of establishment and mutation are dependent on the mechanisms of the virus and cannot be changed by human intervention. The importance of improving biosecurity on farms in order to reduce the probability of spread is therefore stressed from these results.

### Other Considerations

These results show the large influence people who are not farm workers but regularly visit the farm have on the probability of spread. Such people include egg pallet and tray collectors and bird pick up crews. Consultation among different industry bodies is important to emphasize shared responsibility and agreement to biosecurity codes and guidelines. Further training for both farm workers and people who visit farms in regard to the importance of biosecurity is always beneficial. The integrated nature of the Australian chicken meat industry by a small number of companies allows for this shared responsibility and relative ease of communication across a range of networks. However, this may well be lacking in the Australian layer sector due to the nature of this industry which has a high level of numerous, privately owned farms (55). As new information arises related to the volume and frequency of spread pathways that occur in the Australian commercial chicken industry, as well as further information on the behavior and mechanisms of the AI virus, these can be used to update the input parameters in the spread scenario tree models.

Detection and reporting was not highly influential in this model as this node simply represented the proportion of farms that would detect or report at a relatively fixed detection threshold. However, this study did indicate that spread between sheds is likely to have already occurred before detection. Other factors related to detection and reporting were not assessed and should be considered for future studies, particularly for high-risk farms. These include those factors assessed in previous modeling studies such as; the impact of lowering the detection threshold, frequent sampling of farms considered high risk, and ensuring prompt action after detection (9–11). Frequent sampling can improve knowledge of LPAI transmission which has been demonstrated to be largely unknown in this study particularly in the Australian context. AI surveillance in poultry in Australia is currently not supported by the industry due to the consequences outlined in the AUSVETPLAN associated with H5 or H7 detections (56). Performance of surveillance in some form, such as sampling sentinel flocks or poultry at slaughter and processing should be considered for the Australian poultry industry (38).

Given this model considers and follows the probabilities of exposure quantified by Scott et al. (1), the probabilities estimated in this study can be considered representative for the same region as that of Scott et al. (1); the Sydney basin region. Extrapolating these results to other regions, poultry species or non-commercial chicken farms must be done with caution as differences in the probabilities of exposure may exist. However, the framework of this model can be used to aid in the development of similar risk assessment models for these different farms.

## Conclusion

This study indicates that the probability of no establishment is the most likely end-point after exposure and infection of LPAI in one chicken. Nodes linked to attributes of the virus, such as the probability of establishment and the probability of mutation, were the most influential factors impacting the probability of LPAI and HPAI spread, respectively. While these cannot be changed by human intervention, some on-farm actions can be performed to potentially reduce the probability of spread. Biosecurity and cleanliness on farms, with particular attention to equipment and egg trays between sheds and farms, respectively, as these were found as the most likely spread pathways, will reduce the probability of spread. The results of this study and that of the exposure risk assessment in Scott et al. (1) help estimate the overall probability of spread and spread pathways of LPAI and HPAI in Australian commercial chicken enterprises. The results also provide guidance to the Australian commercial chicken industry on the importance of farm workers and people who regularly visit farms in performing biosecurity practices, as this is part of a shared responsibility in safeguarding the industry against AI.

## Ethics Statement

This study was carried out in accordance with the recommendations of the Human Ethics Committee of the University of Sydney, Australia with written informed consent from all subjects. The protocol was approved by the the Human Ethics Committee of the University of Sydney, Australia (ethics reference number: 2015/252).

## Author Contributions

The first author AS was involved with investigation, methodology, writing of the original draft and reviewing and editing. J-AT formed initial conceptualization of the study, and was involved with formal analysis, methodology, project administration, supervision of AS, and reviewing and editing the manuscript. MS was also involved in investigation, methodology, project administration, supervision of AS, and reviewing and editing the manuscript. BB and KG were also involved with initial conceptualization of the study, formal analysis, methodology and provided reviewing and editing. BM, AB and PG were involved with conceptualization, project administration funding/support of the study. PG also provided methodology and supervision. BM provided reviewing and editing. MH-J was heavily involved in formal analysis of the results, conceptualization of the study, methodology, project administration, supervision of AS, and reviewing and editing the manuscript.

## Conflict of Interest Statement

The authors declare that the research was conducted in the absence of any commercial or financial relationships that could be construed as a potential conflict of interest.
