# Supplementary material for: Low- and High-Pathogenic Avian Influenza H5 and H7 Spread Risk Assessment Within and Between Australian Commercial Chicken Farms
Source: Front Vet Sci. 2018 Apr 9;5:63. doi: 10.3389/fvets.2018.00063 (PMC5900437; doi:10.3389/fvets.2018.00063)
Supplement: Supplementary file 1 [file data_sheet_1.docx]

Supplementary Material

Low and high pathogenic avian influenza H5 and H7 spread risk assessment within and between Australian commercial chicken farms

**Angela Bullanday Scott, Jenny-Ann LML Toribio, Mini Singh, Peter Groves, Belinda Barnes, Kathryn Glass, Barbara Moloney, Amanda Black, Marta Hernandez-Jover***

*** Correspondence:** Marta Hernandez-Jover: Charles Sturt University, Graham Centre for Agricultural Innovation; School of Animal and Veterinary Sciences, Australia. Tel: +61 (0) 2 69332086; fax: +61 (0) 2 69332991; Email address: mhernandez-jover@csu.edu.au (M. Hernández-Jover).

*Type of exposure.* The spread scenario tree begins after exposure to LPAI and this node considers the type of exposure to LPAI i.e. direct or indirect as subsequent infection will depend on how exposure to the virus occurs. For the purpose of this model, direct exposure is defined as physical contact between a wild bird and a commercial chicken or direct contact between a commercial chicken and wild bird faeces. Indirect exposure is defined as contact between a commercial chicken with the virus through a medium i.e. through water, fomites or vectors. Fomites include boots and equipment where wild bird faeces may contaminate these fomites and then be presented to chickens through movement. Vectors may become infected with the virus, most notably insects, mice and rats, and may shed the virus in the presence of chickens or be consumed by chickens; in this case they act as biological vectors. Vectors may also present the virus to chickens through movement only; in this case they act as mechanical vectors.

The probability of each type of exposure differs depending on the farm type. In addition, the probability of infection differs depending on the type of exposure (Yao et al., 2014). Information used to estimate this probability node was from the exposure scenario tree of the respective farm type. The total average probabilities of direct and indirect exposure were calculated from the three seasons for each farm type. The averages were then summed together and then divided by this summation to give a proportion of each exposure for each farm type. The direct and indirect exposure branches of this node lead to the next nodes; infection from direct and indirect exposure respectively. The branches that follow are then identical.

*Infection from direct exposure.* This node estimates the probability of infection after direct exposure to LPAI virus. The branches for this node are yes and no i.e. infection from direct exposure does or does not occur respectively. Information used to estimate this node was acquired from scientific literature. Yao et al. (2014) performed experiments assessing the number of chickens infected from exposure to H9N2 LPAI virus through different routes at various virus concentrations and dilutions. The intranasal, gastrointestinal and aerosol routes were assessed. For this model, intranasal and gastrointestinal routes fall under direct exposure. The gastrointestinal route also falls under indirect exposure as well as the aerosol route. Webster et al. (1976) revealed that the average virus concentration in tracheal swabs from Mallards infected with LPAI H5N2 was 10^3.8^ EID 50/ml over six days post inoculation. This figure was used as the assumed virus concentration during intranasal exposure in realistic scenarios. Yao et al. (2014) exposed 26 and 18 chickens with LPAI H9N2 virus via the intranasal route at concentrations of 10^4.69^ and 10^3.69^ respectively. Of these 44 chickens, 42 became infected at these concentrations. A Beta distribution was created using these figures to account for uncertainties; Beta (43, 3). These concentrations were chosen for this model as they were closest to the assumed virus concentration during intranasal exposure. Webster et al. (1976) also revealed that the average virus concentration in cloacal swabs from Mallards infected with LPAI H5N2 was 10^2.04^ EID 50/ml over five days post inoculation. This was assumed as the average virus concentration present in infected duck faeces. However it is appreciated that virus concentration during direct exposure via the gastrointestinal route can range significantly. This is due to factors including the amount of faeces present in the environment and the amount consumed by the chicken. Yao et al. (2014) exposed chickens to the LPAI H9N2 at various concentrations via the gastrointestinal route. The virus concentration 10^2.69^ EID 50/ml was the closest virus concentration used in this study to Webster et al. (1976)’s average of 10^2.04^ EID 50/ml. At this virus concentration and route, 22 chickens were exposed and one was infected therefore providing a proportion of 0.045. A Pert distribution was created ranging from 0 to 1 with the most likely at 0.045; Pert (0, 0.045, 1) to account for the large range of virus concentration. Selleck (2015) revealed that two out of two in-contact chickens housed with chickens infected with LPAI H5N3 became infected with the virus. A Beta distribution was created using these figures; Beta (3, 1). The final probability for the ‘yes’ branch of this node was created by averaging the two Beta distributions and Pert distribution created from scientific literature. The ‘no’ branch was then simply one minus this value. The focus of this model is on virus subtypes H5 and H7. However *s*cientific literature assessing exposure and infection to LPAI H5 or H7 virus in chickens was lacking. Therefore it is assumed the study by Yao et al. (2014) on LPAI H9N2 can be applied to LPAI H5 and H7.

*Infection from indirect exposure.* This node estimates the probability of infection after indirect exposure to LPAI virus. The branches for this node are yes and no i.e. infection from indirect exposure does or does not occur respectively. Information used to estimate this node was acquired from scientific literature. As mentioned, the aerosol and gastrointestinal routes are classed as indirect exposure for this model. Jonges et al. (2015) sampled air surrounding LPAI infected commercial poultry farms. Viral loads from positive samples were relatively low. In addition, no positive samples were detected in air surrounding 83 LPAI infected swans. Yao et al. (2014) experimentally exposed chickens to various virus concentrations of LPAI H9N2 via the aerosol route. It is assumed in realistic scenarios the virus concentration during aerosol exposure is very low. Therefore the lowest aerosol concentration used by Yao et al. (2014) was selected. This concentration was 10^2.69^ EID 50/ml; where out of 10 exposed chickens, none became infected. A Beta distribution was created from these figures to account for uncertainty; Beta (1, 11). Indirect exposure via the gastrointestinal route is assumed at lower concentrations than direct exposure. The lowest two virus concentrations used by Yao et al. (2014) were therefore selected. These concentrations were 10^2.69^ and 10^1.69^ EID 50/ml where in total, 53 chickens were exposed and one became infected. A Beta distribution was created using these figures; Beta (2, 53). The likelihood of aerosol exposure compared to indirect gastrointestinal exposure was also considered. Using the exposure tree of the respective farm type, the probability of aerosol exposure was summed together for all three seasons as well as the probability of indirect gastrointestinal exposure. These two summations were then summed together and then divided by the total to give a proportion of likelihood for each route. These likelihood proportions were then multiplied by their respective Beta distribution i.e. aerosol or indirect gastrointestinal exposure. The final value for the ‘yes’ branch for this node was the sum of these two multiplications. The final value therefore considers both the likelihood of the route occurring as well as the probability of infection via that route.

*LPAI subtype can spread among chickens.* This node follows from the ‘yes’ branch of LPAI infection from direct or indirect exposure and estimates the probability the LPAI subtype that has infected one chicken is a subtype that can spread to other chickens. The branches for this node are yes and no i.e. the LPAI subtype can or cannot spread to other chickens. If no, the pathway leads to no establishment of the virus. If yes, the pathway continues to the next node; establishment of LPAI. Information used to estimate this node was from world outbreak data retrieved from FAO EMPRES-i (2016). This data reports all H5 and H7 LPAI and HPAI detected in poultry across the globe since 2004. It was used to count the number of outbreaks for each H5 or H7 subtype; there are nine types of neuraminidase (N) for each H5 and H7 subtype. It is assumed these reported outbreaks are H5 or H7 virus subtypes that are able to spread amongst poultry since the outbreaks caused enough changes within poultry to be able to be detected and reported. The data was filtered to obtain outbreak information from chickens only. Both LPAI and HPAI were considered however efforts were made to only consider novel introductions from wild birds with subsequent outbreaks and not those reported from spread of the virus from infected farms. Therefore outbreaks excluded include post-2003 H5 HPAI reports from Asian H5N1 endemic countries and post-2014 reports of HPAI H5N1/N2/N8 from Europe and North America as these reported outbreaks were likely due to extensive HPAI spread. In total, nine of 18 H5 and H7 subtypes were counted. A Beta distribution was created using these figures to account for uncertainty; Beta (10, 10). This node therefore estimates that approximately half of all H5 and H7 LPAI subtypes that are exposed to a chicken will spread, regardless of the specific N type.

*Establishment of LPAI after infection in one chicken.* This node estimates the probability that the LPAI virus will become established in the flock after infection in one chicken. Information used to estimate this node was derived from the probability of infection dying out after exposure occurred (Probability of extinction); with the probability of establishment being 1- probability of extinction. This probability of extinction of infection was calculated with a Poisson branching process which used a range of different R as the mean of the Poisson distribution and a serial interval of 3.5 days. A serial interval is the time between successive cases of an infectious disease (Fine, 2003). Estimates of R were produced by fitting a discrete-time model to daily deaths from sheds using data from the 2013 HPAI outbreak in Australia. According to this model, the mean of the R of the HPAI virus causing this outbreak was estimated around 1.41 and 1.57. There is a high degree of uncertainty in estimating the R of LPAI and it may or may not be similar to the R of HPAI (Gonzales et al., 2011). For the purpose of this model, the probability of establishment for a LPAI virus with an R between 1.3 to 1.4 was used, and incorporated into the model with a uniform distribution (Uniform(0.423,0.511)).

*LPAI subtype leads to clinical signs in chickens after infection.* This node estimates the probability that clinical signs will be displayed by chickens infected with an LPAI subtype that can spread amongst chickens. This node considers the fact that some LPAI infections cause little or no clinical signs in chickens (Swayne, 2008). The branches for this node are yes and no i.e. the LPAI subtype does or does not lead to clinical signs in chickens. Information used to estimate this node was acquired from scientific literature. Spickler et al. (2008) reviewed the literature listing studies that assessed morbidity or mortality in chickens infected with AI virus. LPAI H5 and H7 subtypes were counted from this review and it was found 18 of 40 subtypes caused either morbidity or mortality in chickens. In addition, Spackman et al. (2010) experimentally infected chickens with 12 LPAI H7 subtypes; some subtypes were the same but were from different origin species. Of those 12, six lead to clinical signs in chickens. Both papers report an onset of clinical signs of as little as 1-2 days. These two papers were combined where of 52 LPAI H5 and H7 subtypes, 24 caused clinical signs in chickens. A Beta distribution using these figures was created to account for uncertainty; Beta (25, 29).

*Proportion of chickens that show clinical signs from LPAI infection.* This node estimates the proportion of LPAI infected chickens within a flock that will display clinical signs. This node assumes the LPAI subtype established within the flock is a subtype that can both spread amongst chickens and can produce clinical signs as estimated in the previous nodes. The branches for this node are yes and no i.e. chickens do or do not display clinical signs. Information used to estimate this node was obtained from scientific literature. Jones and Swayne (2004) and Mo et al. (1997) experimentally inoculated chickens with LPAI H7N3 and H5N2 viruses respectively and counted the number of chickens that displayed clinical signs. The two studies combined revealed out of a total of 23 chickens, six displayed clinical signs. A Beta distribution was created using these figures to account for uncertainty; Beta (7, 18).

*LPAI detection and reporting.* This node estimates the probability that after LPAI is established within a flock of chickens that it will be detected and reported to the appropriate authorities by the farmer. Information used to estimate this node was derived from the survey on commercial layer and meat chicken farms by (Scott et al., 2018b). In this survey farmers were asked to report signs in chickens that they would consider unusual and prompt them to contact someone. The total number of signs was determined per farm type. Of these signs, those that were strongly attributable to LPAI were counted separately. These signs were a drop in egg production, respiratory signs, mortalities, lethargy, lacrimation or other eye signs and gastrointestinal signs (Barnes, 2009). A Beta distribution was used using the number of answers attributable to LPAI (s) and the total number of answers (n) and was used as the probability for the ‘yes’ branch. This was done for each farm type and therefore five beta distributions were created. If the ‘yes’ branch is followed, the end-point of ‘limited LPAI spread’, as described above, is reached. If the ‘no’ branch is followed, the next node ‘LPAI spread methods shed-shed’ or ‘LPAI spread methods farm-farm’ is reached.

*Mutation of LPAI to HPAI.* This node estimates the probability of mutation from LPAI to HPAI. Information used to estimate this node was obtained from an expert opinion workshop held in November 2015 (Singh et al. 2016). A modified Delphi method was used in the expert opinion workshop (Custer et al., 1999; McBride et al., 2003; Vose, 2008; Speirs-Bridge et al., 2010). The workshop initially involved experts responding to a questionnaire sent through email followed by a face-to-face workshop. During this workshop, probability estimates resulting from the questionnaire were anonymously presented to workshop participants for discussion. Experts were then requested to review and revise their answers if they saw fit using a final paper questionnaire. Only the second probability estimates were considered. Ten experts attended the face-to-face workshop and expertise in topics ranged from wild bird behaviour, virology and the poultry industry. The question asked to the experts to estimate the probability of this node was; “Imagine 100 sheds each of the following operation types where LPAI has recently been established. In how many of these sheds would LPAI mutate to HPAI?” Experts were asked to give the lowest, most likely and highest estimate of mutation and the question was asked for each farm type. Experts were also asked to rate how confident (50 to 100%) they were that the true value would fit within their interval. Assumptions when providing estimates for this node include; H5 and H7 are the only subtypes considered, answers were over one production cycle hence the estimate is low for meat chickens compared to layers, density of birds in a shed has a significant role, and there is no restriction in horizontal contacts on site. Each answer was converted to 80% derived intervals using LogNormal transformation on Microsoft Excel (PC/Windows 7, 2010) in order to normally distribute the experts’ estimates. Pert distributions were created from the converted answers of each of the ten experts. Discrete distributions were then used to combine all expert answers for the question per farm type. The discrete distribution enabled weightings of answers, where experts could be weighted depending on their level of expertise in certain topics. Weightings were low, medium and high; 1 being low and 3 being high. Virologists had heavier weightings compared to other experts for this question.

*LPAI spread methods shed to shed.* This node estimates the probability that LPAI will spread via specific pathways from one shed to another on the same farm. The branches for this node are the pathways considered for shed to shed spread; boots or clothing, vermin such as mice or insects, other animals such as dogs and cats, aerosol and movement of equipment between sheds. This node uses combined information from the survey by Scott et al. (Scott et al., 2017; 2018b) and also on scientific literature reporting LPAI virus survival and presence on particular materials and fomites (Tiwari et al., 2006; Wood et al., 2010; Achenbach and Bowen, 2011; Nielsen et al., 2011; Jonges et al., 2015). This node does not explicitly consider the volumes and frequency of the spread pathways, and instead it considers the proportion of farms that perform or have these pathways present in combination with the survival of the virus on these pathways. In the survey by Scott et al. (Scott et al., 2017; 2018b), farms were visited and the following answers respective of each pathway were recorded; the use of foot baths, the presence of mice/rats and/or insects inside sheds, whether or not farm cats and dogs were allowed on the shed, the distance between sheds, and whether or not disinfection of equipment occurs between sheds. For the distances between sheds, the number of farms that had sheds less than 150 metres from each other were recorded simply because Jonges et al. (2015) had sampled air at 150m and lower and this study was to be used in this node. Values are listed in Table 1 for each farm type. A Beta distribution was created for each of the answers of the respective pathways and this was performed per farm type. The pathways boots or clothing, equipment and other animals transfer the virus mechanically. Literature was searched to find the length of virus survival on different materials. Tiwari et al. (2006) demonstrated that virus survival was at least one day on a variety of material, including three days on gumboots, steel and plastic. AI virus survival was found to be two days in chicken faeces (Wood et al., 2010). Therefore assuming these three pathways occur every day, the probability that the virus will survive when carried by these pathways is one.

AI virus isolation in vermin has been studied to determine their importance in spread during outbreaks. It has generally been concluded that mice and rats do not play significant roles in the spread of AI virus but insects may (Achenbach and Bowen, 2011; Nielsen et al., 2011). Rats were inoculated with LPAI virus and zero of 12 rodents were positive upon virus isolation in one study (Achenbach and Bowen, 2011). Nielsen et al. (2011) conducted an experiment in which flies were fed H7N1 and H5N7 LPAI of three different two-fold virus dilutions. Groups of flies fed the same subtype and dilutions had their contents pooled. A total of 171 fly pools were examined, and of those 73 were positive for the virus (Nielsen et al., 2011). A Beta distribution using both these studies was created using 183 as (n) and 73 as (s). This Beta distribution was multiplied with the Beta distributions of vermin answers from the survey data for each farm type.

The survival of LPAI in the air was estimated using the study by Jonges et al. (2015) which involved the collection of air samples surrounding LPAI infected birds. All procedures were performed in triplicate. Samples at three distances under 150m at different bearings from LPAI H7N7 infected chicken farm were collected; the distances were 42m, 53m and 150m. No virus was detected in any of these samples. A Beta distribution was created (Beta(1,10)) and this was multiplied by the Beta distributions of the number of farms with sheds less than 150m from each other for each farm type.

The Beta distributions created from survey results for boots or clothing, equipment and other animals were simply multiplied by one. For each farm type, the multiplied Beta distributions were then all summed together. Each multiplication was then divided by this summation to give a proportion for each pathway and to ensure the sum of all pathways was one. These final values were used as the final probabilities of each pathway. The final values therefore consider both the likelihood of the pathway occurring as well as the probability of survival of the virus when travelling via that pathway.

*LPAI spread methods farm to farm.* This node estimates the probability that LPAI will spread via specific pathways from farm to farm. Information used to estimate this node was derived from the expert opinion workshop held in November 2015 (Singh et al. 2017b). The question asked to experts to estimate this node was “Imagine 100 LPAI established (farm type) farms. Realistically how many of these will experience LPAI spread to at least one other chicken farm through each of the following pathways?” The farm to farm pathways considered in this node were aerosol, infected wild bird going from one farm to another, other animals including vermin and farm cats and dogs, new bird delivery transport, bird pick up transport both live and dead, feed delivery transport, manure collection, farm workers, trades people such as electricians and plumbers, shared equipment between farms, egg trays and egg pallets. The latter two pathways were applied to layer farm types only. The experts can be assumed to have considered volume, frequency and survival of the virus via these pathways in their answers. The same methodology in using the experts’ answers as described for the node ‘Mutation of LPAI to HPAI’ was applied to this node. This question was asked for each farm type. The following table provides the distributions used for each pathway for each farm type (median (5 – 95%), where the sum of the distributions of the pathways for each farm type was 1.

| - Spread pathway | - Non free range meat chicken | - Free range meat chicken | - Cage layer | - Barn layer | - Free range layer |
| --- | --- | --- | --- | --- | --- |
| Aerosol | - 0.058 (0 – 0.25) | - 0.039 (0 – 0.17) | - 0.029 (0 – 0.20) | - 0.030 (0 – 0.20) | - 0.033 (0 – 0.19) |
| Infected wild bird | - 0.028 (0 – 0.95) | - 0.042 (0.01 – 0.17) | - 0.017 (0.0011 – 0.047) | - 0.019 (0.0014 – 0.13) | - 0.023 (0.0063 – 0.13) |
| Animals (vermin and pets) | - 0.029 (0 – 0.12) | - 0.063 (0 – 0.28) | - 0.025 (0 – 0.18) | - 0.033 (0 – 0.20) | - 0.042 (0 – 0.23) |
| Bird delivery transport | - 0.034 (0.0017 – 0.16) | - 0.025 (0.0033 – 0.16) | - 0.036 (0.0035 – 0.14) | - 0.058 (0.0036 – 0.16) | - 0.032 (0 – 0.16) |
| Bird pick up transport (live and dead) | - 0.25 (0.082 – 0.45) | - 0.25 (0.11 – 0.45) | - 0.10 (0.044 – 0.25) | - 0.10 (0.042 – 0.25) | - 0.10 (0.042 – 0.24) |
| Feed delivery transport | - 0.10 (0.0051 – 0.20) | - 0.079 (0.030 – 0.20) | - 0.057 (0.014 – 0.15) | - 0.054 (0.014 – 0.14) | - 0.054 (0.013 – 0.13) |
| Manure collection | - 0.092 (0.028 – 0.23) | - 0.092 (0.025 – 0.25) | - 0.061 (0.0081 – 0.14) | - 0.064 (0.0071 – 0.14) | - 0.061 (0.0075 – 0.14) |
| Farm workers | - 0.091 (0.0096 – 0.29) | - 0.069 (0.0080 – 0.25) | - 0.092 (0.022 – 0.26) | - 0.087 (0.022 – 0.26) | - 0.087 (0.028 – 0.27) |
| Trades people | - 0.092 (0.030 – 0.21) | - 0.083 (0.016 – 0.21) | - 0.075 (0.013 – 0.19) | - 0.076 (0.015 – 0.18) | - 0.078 (0.014 – 0.18) |
| Shared equipment | - 0.097 (0.017 – 0.22) | - 0.084 (0.014 – 0.20) | - 0.059 (0.015 – 0.19) | - 0.051 (0.014 – 0.11) | - 0.056 (0.013 – 0.11) |
| Egg trays |  |  | - 0.18 (0.084 – 0.31) | - 0.18 (0.079 – 0.31) | - 0.18 (0.081 – 0.30) |
| Egg pallets |  |  | - 0.12 (0.032 – 0.26) | - 0.12 (0.034 – 0.26) | - 0.12 (0.035 – 0.24) |

*HPAI clinical signs, detection and reporting.* This node estimates the combined probability that chickens infected with HPAI will show clinical signs and farmers will detect and report the disease to appropriate authorities. Information used to estimate this node was derived from work conducted by Selleck (2015). In these experiments, chickens were inoculated with HPAI H7 viruses, the majority of which were Australian lineages, and the number of chickens that showed clinical signs recorded. All chickens out of a total of 52 chickens demonstrated clinical signs during infection with an onset of as little as one day. A Beta distribution was created using these figures; Beta (53, 1). This resulting Beta distribution had a probability close to one. The probability of detection is assumed to be very high in this scenario due to the high probability of clinical signs. Therefore no information was used to estimate the probability of detection as a Beta distribution close to one was regarded as accurate for the ‘yes’ branch of this node. If the ‘yes’ branch is followed, the end-point of ‘Limited HPAI spread’, as described above, is reached. If the ‘no’ branch is followed, the next node ‘HPAI spread methods shed-shed’ or ‘HPAI spread methods farm-farm’ is reached.

*HPAI spread methods shed to shed.* This node estimates the probability that HPAI will spread via specific pathways from shed to shed. The branches and methods to calculate their probabilities are the same as the node ‘LPAI spread methods shed to shed’ except for two differences. Namely, the Beta distributions calculating virus survival in vermin and air were different. For vermin, the studies by Sawabe et al. (2009) and Nettles et al. (1985) were used. Sawabe et al. (2009) exposed flies to HPAI H5N1 and then attempted to isolate the virus from the internal organs. Of 59 tested flies, 41 were positive for virus isolation. During a HPAI H5N2 outbreak, rats and mice found inside the farm sheds were sampled. Of 516 rats and mice sampled, zero were positive for virus isolation (Nettles et al., 1985). A Beta distribution was created using both studies (Beta (42, 485)) and this was the Beta distribution used in estimating HPAI virus survival in vermin. During the 2015 HPAI outbreak in the United States of America (USA), air samples surrounding HPAI infected chicken farms were tested (McCluskey, 2015). Of 90 samples tested at a distance of 5m from affected shed, 22 were positive for the virus. A Beta distribution was created using this outbreak data (Beta (23, 69)). These two Beta distributions estimating virus survival in vermin and air were then used in the same methods as described in the node ‘LPAI spread methods shed to shed’.

*HPAI spread methods farm to farm.* This node estimates the probability that HPAI will spread via specific pathways from farm to farm. Information used to estimate this node was derived from the expert opinion workshop held in November 2015 (Singh et al. 2017b). The question asked to the experts was the same question as stated in the node ‘LPAI spread methods farm to farm’, the only difference was that LPAI was replaced with HPAI; “Imagine 100 HPAI established (farm type) farms. Realistically how many of these will experience HPAI spread to at least one other chicken farm through each of the following pathways?” The same methodology in using the experts’ answers as described for the node ‘Mutation of LPAI to HPAI’ was applied to this node. This question was asked for each farm type. The following table provides the distributions used for each pathway for each farm type (median (5 – 95%), where the sum of the distributions of the pathways for each farm type was 1.

| - Spread pathway | - Non free range meat chicken | - Free range meat chicken | - Cage layer | - Barn layer | - Free range layer |
| --- | --- | --- | --- | --- | --- |
| Aerosol | - 0.085 (0.0085 – 0.31) | - 0.075 (0.016 – 0.36) | - 0.048 (0.0052 – 0.30) | - 0.034 (0.0054 – 0.13) | - 0.034 (0.012 – 0.30) |
| Infected wild bird | - 0.038 (0.0080 – 0.14) | - 0.060 (0.010 – 0.18) | - 0.015 (0.0018 – 0.062) | - 0.019 (0.0057 – 0.064) | - 0.034 (0.0066 – 0.11) |
| Animals (vermin and pets) | - 0.042 (0 – 0.13) | - 0.041 (0 – 0.14) | - 0.024 (0 – 0.078) | - 0.026 (0 – 0.083) | - 0.038 (0 – 0.18) |
| Bird delivery transport | - 0.019 (0.0019 – 0.11) | - 0.029 (0.0022 – 0.27) | - 0.031 (0.0018 – 0.18) | - 0.025 (0.0040 – 0.15) | - 0.042 (0.0077 – 0.13) |
| Bird pick up transport (live and dead) | - 0.20 (0.042 – 0.44) | - 0.20 (0.039 – 0.44) | - 0.095 (0.022 – 0.27) | - 0.10 (0.018 – 0.28) | - 0.098 (0.032 – 0.26) |
| Feed delivery transport | - 0.081 (0.0033 – 0.30) | - 0.068 (0.0016 – 0.19) | - 0.049 (0.0018 – 0.10) | - 0.046 (0.0021 – 0.11) | - 0.047 (0.0049 – 0.19) |
| Manure collection | - 0.054 (0 – 0.21) | - 0.064 (0 – 0.20) | - 0.068 (0.013 – 0.23) | - 0.052 (0.0095 – 0.17) | - 0.060 (0.021 – 0.15) |
| Farm workers | - 0.10 (0.031 – 0.35) | - 0.079 (0.022 – 0.28) | - 0.082 (0.022 – 0.29) | - 0.087 (0.020 – 0.25) | - 0.099 (0.020 – 0.28) |
| Trades people | - 0.10 (0.011 – 0.30) | - 0.12 (0.018 – 0.29) | - 0.075 (0.0054 – 0.20) | - 0.078 (0.0062 – 0.22) | - 0.064 (0.0082 – 0.17) |
| Shared equipment | - 0.090 (0.028 – 0.27) | - 0.095 (0.025 – 0.25) | - 0.076 (0.0084 – 0.21) | - 0.087 (0.0095 – 0.25) | - 0.080 (0.0076 – 0.22) |
| Egg trays |  |  | - 0.16 (0.061 – 0.33) | - 0.17 (0.063 – 0.37) | - 0.14 (0.051 – 0.33) |
| Egg pallets |  |  | - 0.075 (0.030 – 0.27) | - 0.094 (0.032 – 0.32) | - 0.075 (0.028 – 0.27) |

**
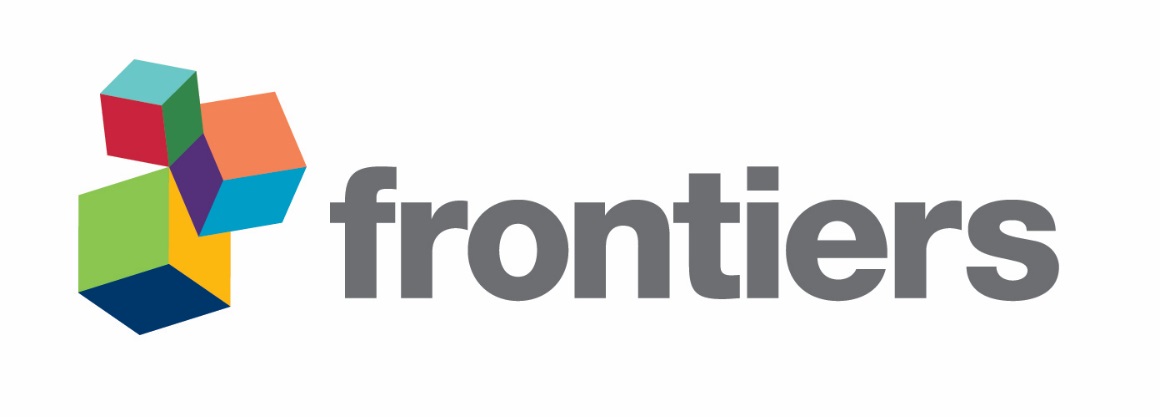
**
